# *C. elegans* SUP-46, an HNRNPM family RNA-binding protein that prevents paternally-mediated epigenetic sterility

**DOI:** 10.1186/s12915-017-0398-y

**Published:** 2017-07-17

**Authors:** Wendy L. Johnston, Aldis Krizus, Arun K. Ramani, Wade Dunham, Ji Young Youn, Andrew G. Fraser, Anne-Claude Gingras, James W. Dennis

**Affiliations:** 1 Lunenfeld-Tanenbaum Research Institute, Mount Sinai Hospital, Toronto, ON Canada; 2 Centre for Computational Medicine, The Hospital for Sick Children, Toronto, ON Canada; 3 Department of Molecular Genetics, University of Toronto, Toronto, ON Canada; 4 The Donnelly Centre, University of Toronto, Toronto, ON Canada

**Keywords:** Germline immortality, Transgenerational sterility, Epigenetic inheritance, HNRNPM, MYEF2, TDP43, P granules, Stress granules, BioID, *C. elegans*

## Abstract

**Background:**

In addition to DNA, gametes contribute epigenetic information in the form of histones and non-coding RNA. Epigenetic programs often respond to stressful environmental conditions and provide a heritable history of ancestral stress that allows for adaptation and propagation of the species. In the nematode *C. elegans*, defective epigenetic transmission often manifests as progressive germline mortality. We previously isolated *sup-46* in a screen for suppressors of the hexosamine pathway gene mutant, *gna-2(qa705)*. In this study, we examine the role of SUP-46 in stress resistance and progressive germline mortality.

**Results:**

We identified SUP-46 as an HNRNPM family RNA-binding protein, and uncovered a highly novel role for SUP-46 in preventing paternally-mediated progressive germline mortality following mating. Proximity biotinylation profiling of human homologs (HNRNPM, MYEF2) identified proteins of ribonucleoprotein complexes previously shown to contain non-coding RNA. Like HNRNPM and MYEF2, SUP-46 was associated with multiple RNA granules, including stress granules, and also formed granules on active chromatin. SUP-46 depletion disrupted germ RNA granules and caused ectopic sperm, increased sperm transcripts, and chronic heat stress sensitivity. SUP-46 was also required for resistance to acute heat stress, and a conserved “MYEF2” motif was identified that was needed for stress resistance.

**Conclusions:**

In mammals, non-coding RNA from the sperm of stressed males has been shown to recapitulate paternal stress phenotypes in the offspring. Our results suggest that HNRNPM family proteins enable stress resistance and paternally-mediated epigenetic transmission that may be conserved across species.

**Electronic supplementary material:**

The online version of this article (doi:10.1186/s12915-017-0398-y) contains supplementary material, which is available to authorized users.

## Background

In 2014, the World Health Organization reported that death from chronic diseases, including cardiovascular disease, cancer, respiratory disease, and diabetes, has surpassed all other causes (http://www.who.int/nmh/publications/ncd-status-report-2014/en/). Approximately 40% of the deaths from chronic disease were premature, with most attributable to environmental stressors, including physical inactivity, obesity, alcohol and tobacco use, and excessive salt intake. Exposure to environmental stressors can cause lasting epigenetic changes in the individual that are transmitted over multiple generations [1]. In a retrospective study of a rural Swedish population it was concluded that famine exposure in the grandfather altered the susceptibility to cardiovascular and metabolic disorders in grandchildren who had not experienced food scarcity [2]. In another example in mice conditioned by foot shock to be adverse to an olfactory cue, the behavior was paternally transmitted to first and second generation progeny [3]. These studies suggest that stress can induce epigenetic changes that can be passed along to subsequent generations, which is a conclusion with significant implications in the prevention and treatment of chronic disease in the 21st century.

During development and throughout an animal’s life, epigenetic changes regulate gene expression, contributing to distinct somatic cell identity and function. Types of epigenetic modifications include DNA methylation and hydroxymethylation, as well as methylation, acetylation, phosphorylation, ubiquination, and O-GlcNAcylation at multiple residues in the exposed tails of histones H2A, H2B, H3, and H4 [4–6]. Regulatory proteins bind to the modified histones causing alterations in chromatin structure and gene expression. At fertilization, totipotency in the zygote is facilitated by genome-wide erasure of most epigenetic markers arising from sperm and oocyte [7]. However, not all epigenetic information is lost. For example, human and *C. elegans* sperm retain some histones that can be transmitted to the zygote [8, 9], and in *C. elegans*, sperm and oocyte transmit PRC2-generated H3K27 methylation, which is important for X chromosome inactivation [10]. Gametes also contribute small non-coding RNA (ncRNA), including miRNA, piRNA, siRNA, and transfer RNA-derived small RNA (tsRNA) [11–13]. Small ncRNA interact with Argonaute (AGO) proteins to target RNA transcripts in the cytoplasm as well as in the nucleus. In the nucleus, ncRNA-AGO complexes bind nascent RNA transcripts and recruit histone- or DNA-modifying enzymes that regulate gene expression [14]. Long ncRNA (lncRNA) are also provided by gametes, and affect gene expression at a number of levels, including regulating histone modification and altering chromatin and higher order chromosome structure [15].

The nematode, *C. elegans*, has a short generation time (3 days at 20 °C) facilitating the study of epigenetic inheritance over multiple generations. During early embryonic development the P4 primordial germ cell divides to generate Z2 and Z3, which give rise to the germline during larval development [16]. Loss of some histone-modifying enzymes results in a progressive mortal germline phenotype, reflecting the importance of histone modifications in epigenetic memory. For example, combined mutation in MET-1 H3K36 and MET-2 H3K9 methyltransferases results in progressive transgenerational sterility [17]. A similar phenotype results from mutations in *spr-5* and *amx-1*, which encode LSD1 H3K4me2 demethylases [18], or SET-2, which encodes an H3K4 methyltransferase [19]. *C. elegans* has a greatly expanded family of AGO proteins, and small ncRNA bound to AGO proteins have been shown to be required for transgenerational epigenetic inheritance [20]. For example, in the germline 21U piRNA bound to PRG-1 AGO silence translation of foreign RNA. Epigenetic memory of the silencing requires the nuclear AGO HRDE-1 [21–23], and HRDE-1 mutants exhibit a progressive transgenerational sterility [23]. Other small ncRNA that participate in epigenetic memory include 26G RNA and their AGO binding partners (ERGO-1 in the soma, ALG-3,4 in the spermatogenic germline), as well as 22G RNA bound by CSR-1 or NRDE-3 [24–27]. Interestingly, small ncRNA-dependent and histone-dependent epigenetic memory often act in concert. For example, piRNAs trigger silencing, but perdurance of the effect depends on H3K9 methylation [21].

In this study, we identify *C. elegans* SUP-46 as an RNA binding protein with homology to human HNRNPM and MYEF2. Using BioID-MS, we identify many RNA granule complex proteins proximal to human HNRNPM and MYEF2, including those of the DROSHA miRNA complex, paraspeckles, the X chromosome inactivation compartment, and stress granules (SG). We show that *C. elegans* SUP-46 localizes to multiple granules, including germline P granules and SG, and is essential for resistance to chronic and acute heat stress. Most remarkably, we uncover an essential function for *C. elegans* SUP-46 in preserving paternally-mediated transgenerational germline immortality. Our findings suggest that HNRNPM family proteins may be important for the paternal transmission of epigenetic memory of stress essential for preserving reproductive potential.

## Results

### *Sup-46* encodes an HNRNPM family protein

**Fig. 1:**
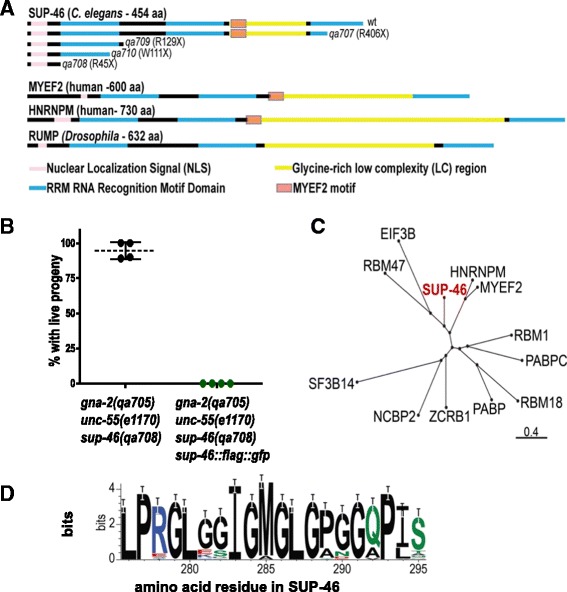
*Sup-46* encodes an RRM protein related to human MYEF2 and HNRNPM, and *Drosophila* RUMP. **a** SUP-46 has a predicted nuclear localization signal, three RRM domains, a glycine-rich low complexity (LC) region, and a MYEF2 motif within the LC region (see Fig. 1d for details of MYEF2 motif). Amino terminus is on the left. The predicted full-length wildtype (wt) or truncated mutant [*sup-46(qa707-qa710)*] proteins are shown. For the mutants, the aa (single letter code) at which the nonsense change (X) occurs is indicated in parentheses. The closest human (MYEF2 and HNRNPM) and *Drosophila* (RUMP) homologs are shown for comparison. **b** SUP-46::FLAG::GFP restores embryonic lethality in adult hermaphrodite *gna-2(qa705) unc-55(e1170) sup-46(qa708)* (assayed by Unc-55 phenotype). The presence of SUP-46::FLAG::GFP was determined by examining for GFP (*green* data points) or no GFP (*black* data points). Hatched line and error bars are the mean ± SD (n = 4 replicate experiments; 20 embryos/replicate). **c** Dendrogram showing the two most closely related human SUP-46 homologs, MYEF2 and HNRNPM. Dendrogram prepared using NCBI BLAST(102) and COBALT Constraint-based Multiple Alignment Tool [110]. Scale shows Grishin protein distance. **d** The MYEF2 motif “LPxGLxxIGMGLGxxGxPI/L/Vx” in MYEF2/HNRNPM proteins, derived using homologs from 61 invertebrate and vertebrate species, listed in Additional file 1: Table S8. Color code for aa: black – non-polar, green – polar, blue – basic, red – acidic

Single-nucleotide polymorphism mapping and high-throughput sequencing of *sup-46(qa707)* genomic DNA were used to identify a mutation causing a premature stop codon in *C25A1.4*[1216C > T (nucleotide number of the spliced coding sequence); amino acid change R406X (stop)]. Independent mutations causing premature stop codons were identified by PCR in the other three mutant alleles, namely *(qa708)*[133C > T; R45X], *(qa709)*[385C > T; R129X], and *(qa710)*[333G > A; W111X], and predicted protein truncations are shown in Fig. 1a. *C25A1.4(RNAi)* suppressed *gna-2(qa705)* embryonic lethality from 100% to 8% (Additional file 1: Table S1), and SUP-46::FLAG::GFP re-established embryonic lethality in *gna-2(qa705)* *sup-46(qa708)* (Fig. 1b), verifying *sup-46* identification.

The closest *Drosophila* and human homologs of SUP-46 are the RNA-binding proteins RUMP, MYEF2, and HNRNPM (Fig. 1a, c). RUMP binds nanos mRNA and the AGO Aubergine to enable posterior accumulation of Nanos protein and anteroposterior polarity in the embryo, and also binds *gypsy* chromatin insulator complex proteins to antagonize *gypsy* function in the nucleus [28–30]. MYEF2 suppresses myelin basic protein expression, and binds the RUNX-1 transcription factor [31, 32]. HNRNPM regulates translation at post-synaptic densities of rat cortical neurons as well as splicing in the nucleus [33, 34]. SUP-46, RUMP, MYEF2, and HNRNPM have the same general architecture, with a nuclear localization signal, three RNA-Recognition Motif (RRM) domains of similar length, and a glycine-rich low complexity (LC) region of variable length between RRMs 2 and 3. The RRM is the most common RNA-binding module, and proteins bearing this motif bind and regulate RNA, and sometimes also DNA [35]. RRMs are often present as multiple copies, which serves to increase specificity and affinity for their short, degenerate RNA target sequences [36–38]. LC regions typically mediate protein-protein binding.

### Human SUP-46 homologs associate with multiple non-coding RNA compartments

RNA typically associates with RNA-binding proteins in protein complexes, some of which phase separate into large microscopically visible RNA granules [39]. To identify RNA-binding protein complexes and granules that might contain SUP-46 family proteins we performed proximity biotinylation coupled to mass spectrometry (BioID-MS) on cells expressing the human SUP-46 homologs, HNRNPM and MYEF2, fused to Bir-A*. BioID is based on utilization of the abortive biotin ligase (BirA*) fused to a protein of interest, allowing in vivo labeling of proximal proteins [40]. The covalent nature of the biotin label and the extreme stability of biotin-streptavidin interaction allow characterization of proteins that localize to compartments that can be refractory to traditional affinity purifications such as membrane, cytoskeletal structures, centrosomes, and RNA complexes [41–44]. Proteomics and statistical analysis of two biological replicate purifications of HNRNPM and MYEF2 (alongside negative controls) identified 133 and 110 high-confidence proximity interactions, respectively (Additional file 1: Table S2). Among these interactions, 72 proximal proteins were identified in both HNRNPM and MYEF2.

**Fig. 2:**
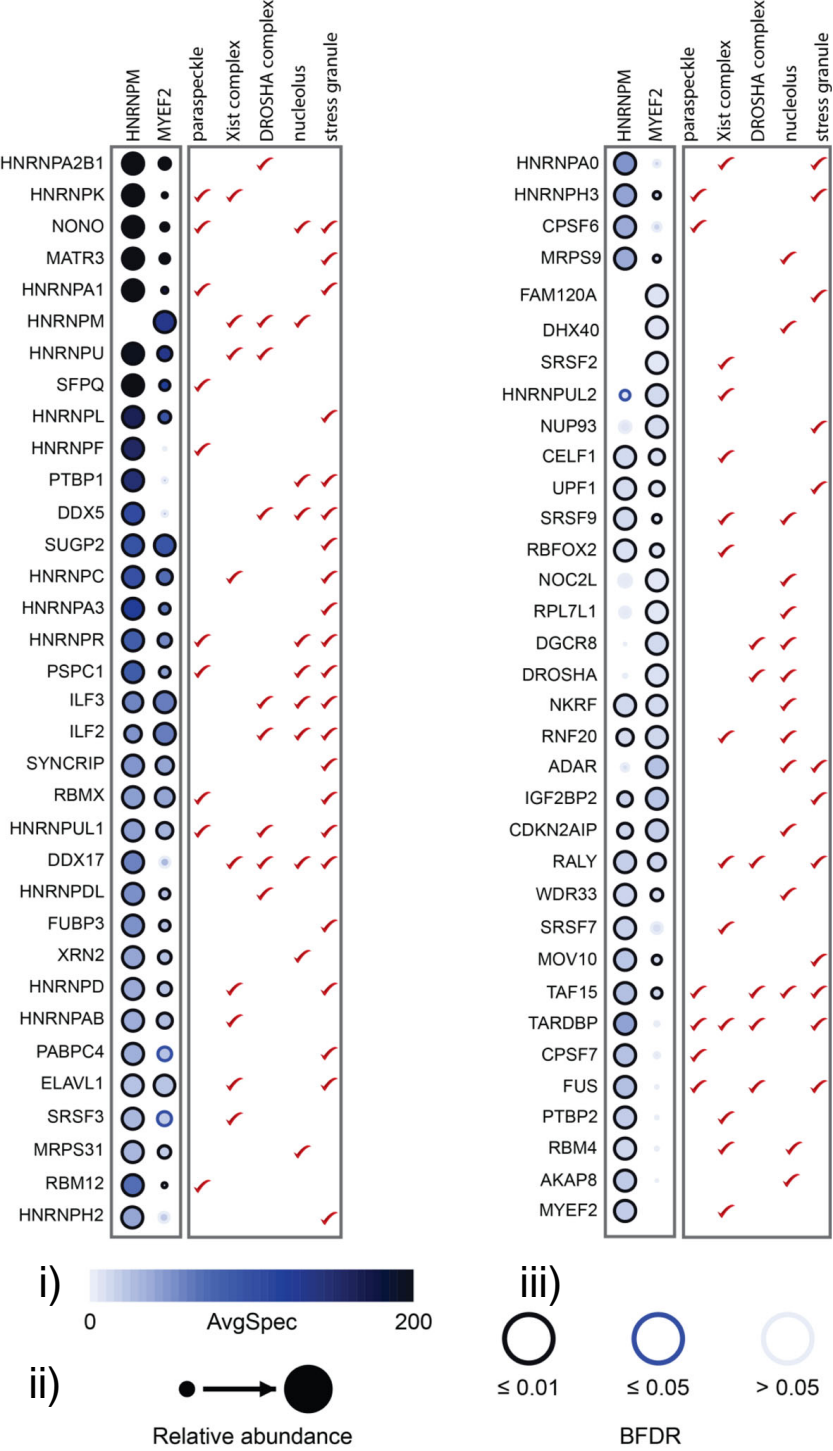
BioID of BirA*-FLAG-tagged HNRNPM and MYEF2 from HEK293 cells highlighting significant groups of preys annotated to be in specific RNA granules or RNA associated complexes. Prey proteins found in different RNA-associated compartments are based on published data: Paraspeckle [45], X chromosome inactivation complex [48], DROSHA microprocessor complex [50–52], Stress granule [53], and Nucleolus (using g:Profiler; http://biit.cs.ut.ee/gprofiler/index.cgi [111]. Data are the mean of two biological replicate experiments. (i) Spectral counts of each prey protein are displayed as a gradient of intensity in *blue* as indicated in the scale. (ii) Relative abundance between HNRNPM and MYEF2.(iii) Bayesian false discovery rate. See also Additional file 1: Tables S3 and S4

Preys identified in HNRNPM and MYEF2 BioID screens included constituents of RNA compartments, including the paraspeckle, the X chromosome inactivation (Xist) complex, the microprocessor/DROSHA complex, SG, and the nucleolus (Fig. 2, Additional file 1: Tables S3 and S4; note that several of these components were identified in more than one of these structures). Sixteen of 40 known paraspeckle proteins [45] were identified proximal to HNRNPM and/or MYEF2, extending earlier data showing that HNRNPM binds to a core paraspeckle component, SFPQ/PSF [46]. Paraspeckles contain the lncRNA NEAT1, and appear to have multiple regulatory roles in the nucleus, including gene expression during stress [47]. Twenty-two of 51 proteins key to the gene regulatory function of the X chromosome inactivation complex [48] were also identified proximal to HNRNPM and/or MYEF2. Recently, HNRNPM and MYEF2 were shown to bind directly to XIST, the lncRNA that localizes to the inactive X chromosome to recruit the polycomb repressive complex, resulting in histone H3K27 methylation and silencing [48, 49]. In addition to paraspeckles and the X chromosome inactivation complex, which contain lncRNA, DROSHA microprocessor proteins were identified. The microprocessor processes and regulates miRNA, and proximal proteins included the microprocessor subunits DGCR8 and DROSHA (MYEF2 only), as well as 13 of 19 accessory proteins [50–52]. HNRNPM and/or MYEF2 proximal proteins also included 34/139 proteins identified in the SG proteome of human U-2 OS cells exposed to sodium arsenite and harvested without formaldehyde cross-linking [53]. SG are reversible cytosolic RNA protein granules that form in response to heat shock and other stressors, and are believed to modulate the stress response [54].

### SUP-46 is present in the nucleus, cytoplasm, and multiple RNA granules

**Fig. 3:**
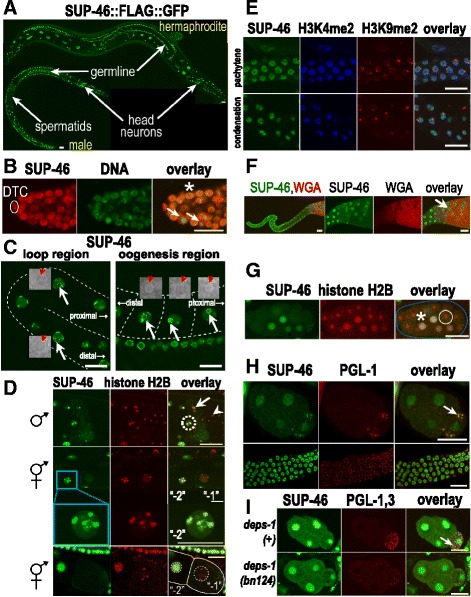
SUP-46 is ubiquitously expressed and localizes to multiple nuclear and cytoplasmic compartments. **a** SUP-46 is nuclear in developing gametes in the germline and in somatic cells (head neurons indicated), but is not detected in spermatids. **b** SUP-46 is nuclear in non-dividing cells (*asterisk*), but localizes to the cytoplasm in dividing cells (*arrows*) in the mitotic region of the adult hermaphrodite gonad. The distal tip cell (DTC) at the distal end of the gonad is indicated. **c** SUP-46 is absent from the nucleolus in germ cells of the loop region of the hermaphrodite germline (*white arrows* left-hand image), but accumulates in the nucleolus of developing oocytes in the oogenesis region (*white arrows*, right-hand image). Nucleoli are indicated by hatched circles and were identified in corresponding light images, shown as enlarged insets with red arrows pointing to the nucleolus. Gonad is outlined by a hatched line. Orientation of the germline (distal or proximal) is indicated. See also Additional files 2 and 3 showing z series through the loop region or oogenesis region, respectively. **d** SUP-46 is associated with chromatin (marked by histone H2B::mCherry) until just before the meiotic divisions. In the top row, SUP-46 in the male germline is strongly associated with chromatin in primary spermatocytes (hatched circle), but accumulates in perichromatin granules (*arrow*) at meiosis anaphase I. SUP-46 is absent from spermatids (*arrowhead*). In row 2, SUP-46 in the hermaphrodite germline is strongly associated with chromatin in the “–2” oocyte but becomes diffusely nuclear and no longer chromatin-associated in the “–1” oocyte (oocyte closest to spermatheca). Row 3 is a higher magnification image of the “–2” oocyte nucleus from row 2 and shows a granular distribution of SUP-46 on chromatin. Bottom row shows SUP-46 is depleted from the nucleus and accumulates in the cytoplasm of the “–1” oocyte just before nuclear envelope breakdown and ovulation. Oocytes are outlined by *solid lines*, with nuclei outlined by *hatched circles*. All images are oriented with the distal region of the germline to the left. **e** Pachytene region (*top row*) and condensation zone (*bottom row*) of the male germline showing SUP-46 associated with active chromatin (marked by histone H3K4me2), but not inactive chromatin (X chromosome; marked by histone H3K9me2). **f** SUP-46 accumulates in the meiotic residual body (*arrow*) during spermatogenesis. Left image is a low magnification view of a dissected male gonad, with the meiotic zone on the right. Remaining images are a higher magnification view of the meiotic region. Wheat germ agglutinin lectin (WGA) marks spermatids. **g** In embryos, SUP-46 is primarily nuclear in interphase cells (*asterisk*) but accumulates in the cytoplasm of dividing cells (*white circle*), recognized by condensed chromatin marked by histone H2B. Embryo is outlined in blue. See also Additional file 4, a video showing SUP-46 re-accumulating in the nucleus after cell division. **h** SUP-46 accumulates in P granules (*arrow*) of the posterior P2 cell of the embryo (*top row*), but not in perinuclear P granules in the adult germline (*bottom row*). **i** P granule (*arrow*) accumulation of PGL-1,3 and SUP-46 is decreased in the *deps-1(bn124)* mutant raised at the non-permissive temperature (24 °C). (**a**, **c**–**i**) Strain expresses SUP-46::FLAG::GFP. **b** Strain expresses SUP-46::FLAG::mCherry. (**d** Rows 1–3) strain also expresses mCherry::H2B to visualize chromatin and GFP::PH(PLC1delta) to help visualize cell boundaries. (**d** bottom row, **g**) Strain also expresses HIS-24::mCherry and mCherry::H2B. (**h**, **i**) P granules in 4-cell embryos were detected with an antibody to (**h**) PGL-1 or (**i**) PGL-1 and PGL-3. Embryos are oriented with the P2 cell on the right. (**a**, **c**, **d**, **g**) Imaged live. (**b**, **e**, **f**, **h**, **i**) Fixed and stained with antibodies or WGA. (**a**–**i**) Scale bars represent 15 μm. With the exception of (**g**), which is a 14-μm thick confocal composite image, images are of a single confocal optical plane

Based on the BioID data of SUP-46 human homologs, we hypothesized that SUP-46 would also localize to multiple RNA granules. We investigated localization of worm SUP-46 in granules and other cellular compartments using strains expressing fluorescently tagged SUP-46 (with 25kB 5′, and 5kB 3′ native sequence). SUP-46 was expressed ubiquitously in germline and somatic cells (Fig. 3a). In the germline, SUP-46 was cytoplasmic in mitotic cells in the distal region, and nuclear in the non-dividing cells of the distal, transition, and meiotic zones (Fig. 3a, b). Within germ nuclei, SUP-46 was present diffusely, and in granules associated with active chromatin, marked by H3K4me2, but not the inactive X chromosome marked by H3K9me2 (Fig. 3c–e). Just before ovulation, SUP-46 exited the nucleus and accumulated in the cytoplasm of the meiotic “–1” oocyte (position relative to spermatheca) (Fig. 3d; bottom row). Similarly, during spermatogenesis SUP-46 was nuclear and chromatin associated during spermatogenesis, but then accumulated in the sperm anucleate meiotic residual body, which captures spermatocyte cytoplasm following completion of meiosis (Fig. 3f). During oogenesis in the diakinesis region, in addition to localizing to chromatin, SUP-46 also accumulated in the nucleolus (until nucleolar breakdown in the “–2” to “–1” oocyte) (Fig. 3c, right-hand image; Additional files 2 and 3 show confocal series through the germline loop region and oogenesis region, respectively). The localization of germline SUP-46 to multiple nuclear compartments, together with its ability to transit between the nucleus and the cytoplasm, indicates that SUP-46 may regulate multiple RNA-dependent processes during gametogenesis.

In the embryo, SUP-46 was primarily nuclear in non-dividing cells and cytoplasmic in dividing cells (Fig. 3g; Additional file 4, which shows SUP-46 moving in and out of the nucleus from the zygote to the four cell stage). In P_1_ and P_2_, which ultimately give rise to the germline during larval development, SUP-46 also accumulated in cytoplasmic granules that were reminiscent of P (germ) granules (Additional file 4 shows SUP-46 in presumptive P granules in live embryos). Immunolocalization of the constitutive P granule protein PGL-1 confirmed that SUP-46 is present in P granules in the embryo (Fig. 3h, top row), but not in the adult (gonad) (Fig. 3h, bottom row). At 24 °C, localization of PGL-1 and PGL-3 required the nematode-specific protein DEPS-1 [55], and DEPS-1 was also required for P granule accumulation of SUP-46 (Fig. 3i).

**Fig. 4:**
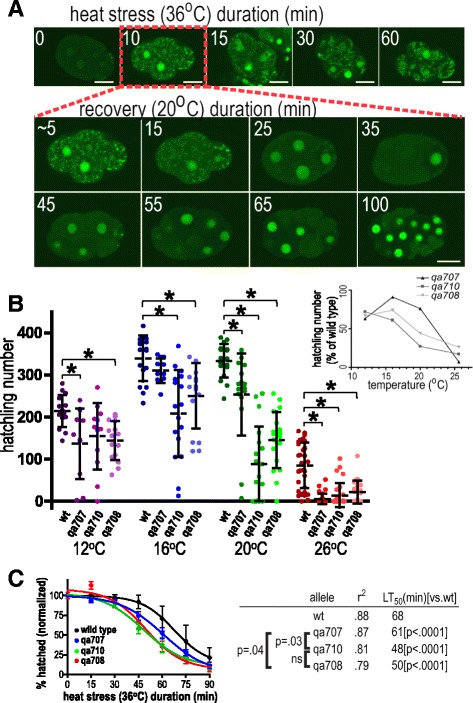
SUP-46 protects from heat stress and localizes to stress granules. **a** SUP-46 accumulates in stress granules and is enriched in the nucleus within 10 min of heat stress, and dissipates from stress granules within 25–35 minutes at 20 °C. Inset numbers are duration of heat stress in individual embryos (*top row*), or recovery in the 10 min heat stressed embryo (*middle* and *bottom rows*). Images are single confocal optical sections of embryos that express SUP-46::FLAG::GFP. Scale bars represent 15 μm. **b** SUP-46 is essential for optimal fecundity, especially at elevated temperature. Data points represent brood counts of individual hermaphrodites. Hatched line and error bars are the mean ± SD (n = 11–35). *Significantly different from wildtype [wt; *sup-46(+)*] at that temperature; *P* < 0.05. Inset shows hatchling number expressed as percentage of wildtype, to illustrate the increased requirement for SUP-46 at 26 °C. **c** *sup-46* mutant embryos have a shorter acute heat stress survival time than wildtype (n = 10–14; 20 embryos/replicate). Error bars are SD. Allelic differences in time to lethality in 50% of the population [LT_50_ (min)] are shown on the right, with shorter heat stress survival indicated by a smaller LT_50_. *P* values for comparison of the different alleles are indicated. ns, not significantly different

Stress can cause RNA and RNA-binding proteins to undergo reversible phase transition into large SG [56]. SUP-46 accumulated in heat stress-induced granules within 10 minutes, and dissipated by 25–35 minutes after heat stress was removed (Fig. 4a). SG identification was confirmed by colocalization with CGH-1 and TIAR-2, which have previously been shown to accumulate in SG (Additional file 5A, B) [57, 58]. SG accumulation was specific for SUP-46, as it was not seen with control GFP strains, including GFP alone, tubulin::GFP, histone H2B::GFP, or EGG-1::GFP (Additional file 5C). In addition to localizing to SG, SUP-46 protein appeared to be increased throughout the cell, including in the nucleus, following heat stress (Fig. 4a). Increased SUP-46 protein could result from increased transcription, increased translation, and/or decreased protein degradation. A related RNA-binding protein (PABP1) was initially decreased during heat shock, but increased by more than two-fold during the ensuing recovery period [59]. In that study, transcript abundance was unaltered, suggesting increased translation as the mechanism. Increased transcription following heat stress has also been reported for genes encoding diverse proteins, including heat shock proteins, transcription factors, signaling molecules, and others [60]. Future studies examining *sup-46* transcript and protein abundance will be helpful to determine the mechanism of increased SUP-46 protein following heat stress.

LC regions in RNA-binding proteins have been shown to regulate SG assembly [61]. Within the LC region of SUP-46, we used NCBI BLAST (https://blast.ncbi.nlm.nih.gov/Blast.cgi?PAGE=Proteins) to identify a novel 20 amino acid sequence that is conserved between *C. elegans* SUP-46 and its human homologs, MYEF2 and HNRNPM. We designated this as the MYEF2 motif, and determined it to be ≥ 70% identical in HNRNPM family members, but not present in other HNRNP proteins (Fig. 1d). To reveal which parts of the SUP-46 protein are required for SG localization, strains were generated with SUP-46 deleted for different parts of the protein and tagged with GFP. All strains had GFP, with reduced (~50%) levels in those deleted beginning after the RRM1 domain, deleted for the LC domain or the MYEF2 motif, or expressing only the LC domain (Additional file 5D). Strains were generated by mos1-mediated single copy insertion with the transgene expressed under the endogenous *sup-46* promoter. Therefore, decreased protein abundance in strains missing the MYEF2 motif suggests a role for the motif in post-transcriptional regulation of the transcript or protein. Following heat shock, robust SG were formed in embryos carrying full-length SUP-46, or SUP-46 deleted for RRM3 or the non-conserved part of the LC domain (Additional file 5E). In contrast, SG were compromised in all three strains missing the MYEF2 motif, or expressing only the LC domain. These results may suggest the MYEF2 motif is necessary, though not sufficient, for robust accumulation of SUP-46 in SG following stress. However, lower protein abundance in strains missing the MYEF2 motif might also explain failed SG formation, since phase separation by LC domains is concentration dependent, at least in vitro [62]. Future studies with human MYEF2 and HNRNPM will be useful to further test the role of the highly conserved, novel MYEF2 motif in transcript/protein stability, and SG formation.

### SUP-46 protects against heat stress

SUP-46 localized to P granules, which are required for resistance to chronic heat stress [63–65], and to SG, which are required during acute heat stress [58]. To test if SUP-46 protects against chronic heat stress, hatchling number (viable offspring) was determined for individual *sup-46* mutant and wildtype hermaphrodites at 12 °C (cold stress), 16 °C and 20 °C (optimal for wildtype), and 26 °C (heat stress). At all temperatures, two or more mutant alleles had fewer hatchlings than wildtype, indicating that *sup-46* is required at all temperatures (Fig. 4b). As a percent of wildtype, mutants produced far fewer hatchlings at 26 °C, indicating that SUP-46 is particularly important during heat stress (Fig. 4b, inset), similar to what has been reported for other P granule proteins. To test for acute heat stress response, survival of comma stage embryos was examined following exposure to 36 °C. Survival was decreased in all three mutant alleles (LT_50_ ≤ 61 min), compared with wildtype (LT_50_ 68 min), indicating SUP-46 is essential during acute heat stress (Fig. 4c). Comparing mutants showed the severely truncated *qa708* and *qa710* alleles had significantly poorer survival (LT_50_ 50 and 48 min, respectively) than the less severely truncated *qa707* allele (LT_50_ 61 min). *Sup-46(qa707)* retains the sequence encoding RRM1, RRM2, and the MYEF2 motifs, which enable SG localization, consistent with the possibility that SG accumulation of SUP-46 contributes to acute heat stress resistance.

We originally identified *sup-46* mutants as suppressors of *gna-2(qa705)* embryonic lethality that depended on glucosamine 6-phosphate N-acetyltransferase-1 (GNA-1) activity [66]*.* Consistent with this finding, the *sup-46* mutant had increased GNA-1 protein expression (~1.4X; Additional file 6a). GNA-2 and GNA-1 (GNPNAT1 in humans) are enzymes in the hexosamine biosynthesis pathway, which ultimately converts fructose 6-phosphate to UDP-GlcNAc. UDP-GlcNAc is a sugar donor in multiple glycosylation pathways, including N-glycosylation and chitin synthesis [67, 68]. Eggshell chitin is critical for multiple early events in *C. elegans* zygotic development, and chitin deficiency is embryonic lethal. *Gna-1* expression is increased following heat stress [69], and UDP-GlcNAc supplied to N-glycosylation extends lifespan and protects against proteotoxic stress in *C. elegans* [70]. In mammalian cells, increased N-glycosylation supports cell surface residency of growth factor receptors and solute transporters that regulate the stress response [71, 72]. These findings suggest that hexosamine biosynthesis pathway stimulation may allow adaptation during stress. Despite having increased GNA-1, the *sup-46* mutant had enhanced, rather than reduced, susceptibility to both chronic and acute heat stress. To examine the relationship between SUP-46 depletion and increased GNA-1, we examined hatchling number in wildtype and *sup-46* mutants raised at 26 °C and fed control [empty vector; *mtv(RNAi)*] or *gna-1(RNAi).* Depletion of either *sup-46* or *gna-1* caused a decrease in hatchlings and the effects were additive, with an average of less than one hatchling detected in *sup-46* mutants also depleted for GNA-1 (Additional file 6b). Therefore, SUP-46 and GNA-1 are each required for heat stress resistance and act at least partially independently of one another.

### SUP-46 controls abundance of sperm and oocyte transcripts

SUP-46 is enriched in the nucleus and encodes an RNA-binding protein, implying it might regulate mRNA abundance and/or splicing. RNASeq was used to compare three biological replicates of wildtype and *sup-46(qa710)* worms raised at room temperature. SUP-46 affected transcript abundance of approximately 11% of genes, with 1479 increased and 844 decreased in the mutant (*P* ≤ 0.05; average 2X change, range 1.2X to 51X; Additional file 1: Table S5). *Gna-1* transcript was increased in the mutant (4.5X; *P* = 4.6 × 10^–23^), consistent with the original isolation of *sup-46(-)* as a GNA-1-dependent suppressor of *gna-2(qa705)* embryonic lethality. In contrast, *sup-46* transcript was decreased (4.2X; *P* = 2.0 × 10^–34^). *Sup-46(qa710)* has a premature stop codon in the second (of 7) exons, predicting transcript degradation by nonsense-mediated decay [73]. Together, the transcript changes detected for *gna-1* and *sup-46* give a high degree of confidence in the RNASeq data.

**Fig. 5:**
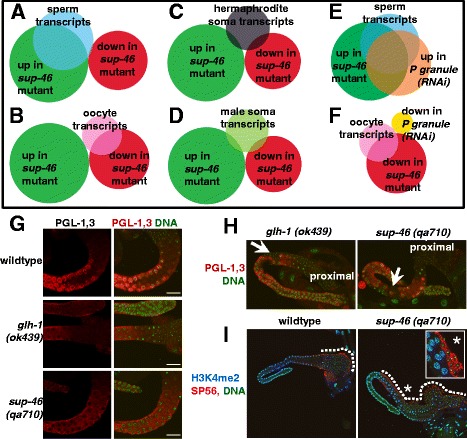
Loss of SUP-46 disrupts P granules, causes ectopic spermatogenesis, and increases spermatogenesis transcripts. **a** 67% (580/872) of spermatogenesis transcripts were increased (*P* = 2.4 × 10^–482^) and < 1% (2/872) decreased (*P* = 4.0 × 10^–14^) in the *sup-46* mutant. **b** In contrast, 51% (180/353) of oogenesis transcripts were decreased (*P* = 3.3 × 10^–156^) and none were increased (*P* = 2.5 × 10^–12^) in the *sup-46* mutant. **c** 3% (14/460) of hermaphrodite soma transcripts were increased (*P* = 7.9 × 10^–5^) and 6% (26/460) decreased (*P* = 0.06) in the *sup-46* mutant. **d** 4% (16/433) of male soma transcripts were increased (*P* = 0.001) and 4% (19/433) decreased (*P* = 0.42) in the *sup-46* mutant. **e** 71% (414/580) of the sperm transcripts that were increased in the *sup-46* mutant were also increased in *P granule(RNAi)* (*P* = 1.1 × 10^–353^). **f** In contrast, none of the oocyte transcripts that were down in the *sup-46* mutant were also down in *P granule(RNAi)* (*P* = 5.0 × 10^–4^). **g** PGL-1,3 were clustered in perinuclear granules in the germline of wildtype males, but diffusely distributed in *glh-1* and *sup-46* mutants. **h** *glh-1* and *sup-46* mutants had ectopic sperm (*arrow*) interspersed with pachytene nuclei in the middle region of the germline. The proximal germline, to which sperm are normally confined, is indicated. **i** Immunolocalization with the sperm-specific antibody, anti-SP56, confirms identification of sperm in the middle region of a *sup-46* mutant male. Regions containing sperm are outlined with a hatched line. The inset (enlarged) shows sperm marked by SP56, and is a different focal plane of the area marked by the asterisk. In wildtype, sperm were restricted to the proximal region, while the *sup-46* mutant had sperm extending as far back as the loop region. (**a**–**f**) Venn diagram circle sizes and overlaps are proportional to the number of genes in each class. Sperm and oocyte transcripts are the overlapping sets of [75] and [74], soma transcripts are from [75], and *P granule(RNAi)* are from [76]. *P* values were calculated using the online hypergeometric distribution calculator, (http://systems.crump.ucla.edu/hypergeometric/index.php). (**g**–**i**) Germlines from male worms were fixed and stained with antibodies and PicoGreen (DNA). Scale bars represent 15 μm. Images are of a single confocal optical plane

*Sup-46* mutants have a reduced number of progeny, hinting that transcripts important for reproduction might be misregulated. To test this hypothesis, SUP-46-regulated transcripts were compared with those enriched in spermatogenesis and oogenesis [74, 75]. We determined that 67% (580/872) of sperm transcripts were increased in the *sup-46* mutant, and less than 1% (2/872) were decreased (Fig. 5a). In contrast, 51% (180/353) of oocyte transcripts were decreased, and none were increased (Fig. 5b). Somatic [75] transcripts in hermaphrodite and male were largely unchanged (Fig. 5c, d). Thus, SUP-46 negatively regulates approximately two-thirds of male gametogenesis transcripts, and positively regulates approximately half of female gametogenesis transcripts.

Depletion of P granule components has previously been shown to increase sperm transcripts [76]. We found extensive overlap of P granule and SUP-46-regulated sperm transcripts, (71%; 414/580) (Fig. 5e), suggesting that SUP-46 might be required for P granules, at least during spermatogenesis. To test this possibility, we examined distribution of PGL-1 and PGL-3, in male *sup-46(qa710)* and *glh-1(ok439)*. GLH-1 is a germline specific DEAD-box RNA helicase related to DDX4, and *glh-1(ok439)* has dissipated P granules at room temperature [77]. Compared with control, *sup-46* and *glh-1* mutants had a significant increase in the number of germlines with disrupted PGL-1,3 (71%, *n* = 32 and 43%, n = 38, respectively) (Fig. 5g). Moreover, ectopic sperm were detected in 9% of *sup-46* and 18% of *glh-1* mutants, indicating failure to restrict spermatogenesis to the proximal germline (Fig. 5h, i). These results are consistent with disruption of P granules and ectopic spermatogenesis being responsible, at least in part, for the elevated sperm transcripts in the *sup-46* mutant. However, decreased oocyte transcripts in the *sup-46* mutant are unlikely to result from poor P granules, since P granule depletion does not decrease abundance of oocyte transcripts [76] (Fig. 5f).

In contrast to the widespread effects on transcript abundance, differences in alternative splicing were found for less than 1% of genes (Additional file 1: Table S6), indicating that SUP-46 is not a global regulator of alternative splicing. Interestingly, of the few genes that did show splice differences, almost half are regulated by the RRM protein TDP-1 [78], and splice changes were noted for *tdp-1* itself (Additional file 7A). SUP-46 and TDP-1 interacted [79] and co-localized in somatic nuclei (Additional file 7B). Consistent with the worm data, we found that the human TDP-1 homolog TARDBP/TDP43 interacted with HNRNPM and, to a lesser degree, with MYEF2 (Fig. 2). In humans, *tardbp* mutation is causative in amyotrophic lateral sclerosis, and TARDBP aggregates in multiple neurodegenerative diseases [80, 81]. Accordingly, it will be important to determine if SUP-46, HNRNPM, and MYEF2 play a role in TDP-1/TARDBP physiology or protein stress pathology.

### SUP-46 prevents mating-dependent progressive hermaphrodite sterility

**Fig. 6:**
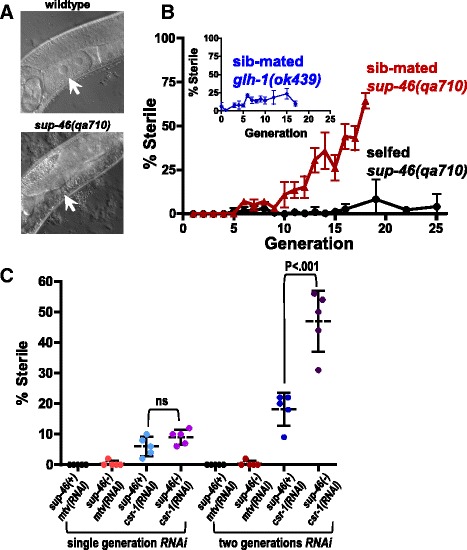
SUP-46 prevents mating-dependent germline mortality in hermaphrodites. **a** Adult *sup-46* mutant hermaphrodites had a defect in oogenesis that developed after multiple generations of mating. In wildtype, oocytes were seen in the proximal germline (*arrow*), while in a *sup-46* mutant mated for five or more generations, oocytes failed to develop (*arrow*). **b** Beginning at generation 5, *sup-46* hermaphrodites mated with sibling males (sib-mated) began to show sterility within the population. Unmated (selfed) *sup-46* hermaphrodites did not exhibit a progressive increase in sterility, and sib-mated *glh-1(ok439)* mutants showed only a very modest increase in sterility. Error bars represent ± SD (*n* = 6). **c** *sup-46* mutant unmated hermaphrodites exposed to *csr-1(RNAi)* for two generations had a large increase in sterility of the population, compared with *sup-46* mutants exposed to control RNAi [*mtv(RNAi)*], wildtype exposed to *csr-1(RNAi*), or mutants and wildtype exposed to *csr-1(RNAi)* for only a single generation. For the second generation, mutant and wildtype hermaphrodites were exposed to *csr-1(RNAi)* in the first generation*.* Hatched line and error bars are the mean ± SD. ns, not significantly different (*P* > 0.05)

*C. elegans* has two naturally occurring sexes, namely self-fertilizing hermaphrodite and male. Male sperm have a competitive advantage over hermaphrodite sperm, resulting in approximately 50% of males in the population following successful mating. Therefore, to keep a ready supply of males, we continuously mated *sup-46* males and hermaphrodites. Over time, we noticed that some hermaphrodites failed to lay any eggs. When viewed under the dissecting microscope, these worms had a characteristic “ventral white patch,” with no embryos in utero. DIC microscopy confirmed the ventral white patches were underdeveloped germlines with few, if any, oocytes (Fig. 6a). Further investigation uncovered a mating-dependent progressive sterility beginning at generation 5, and increasing in penetrance over subsequent generations (Fig. 6b). Unmated (self-fertilizing) hermaphrodites did not show the progressive sterile phenotype, at least up to generation 25. Male *C. elegans* sperm are larger than those of hermaphrodites and, therefore, deliver more cytoplasmic material at fertilization [82]. In mice, ncRNAs contained within the sperm cytoplasm provide critical epigenetic information that communicates details of the ancestral paternal environment to the zygote (reviewed in [83]). Therefore, it is possible that *C. elegans* male sperm deliver more epigenetic information in the form of ncRNA or other epigenetic molecules, than do hermaphrodite sperm.

Loss of the SPR-5 histone H3K4 demethylase enzyme results in progressive sterility and increased spermatogenesis transcripts in selfing hermaphrodites, beginning at generation 15 [18]. Spermatocyte histone H3K4me2 was examined in generation 1 and generation 17 *sup-46* mutant males to determine if failure to erase sperm H3K4 methylation might be responsible for the male sperm-dependent transgenerational sterility. No change was detected in H3K4me2, which typically marks active chromatin, or in H3K27me2me3, which is usually associated with inactive chromatin (Additional file 8). While this finding may suggest SUP-46 depletion causes progressive sterility independently of histone H3 methylation, more in depth studies examining histone modifications at individual genetic loci are needed.

Hermaphrodites that are heterozygous for mutations in the AGO-encoding *csr-1* and *alg-3,4* genes are fertile. In contrast, homozygotes exhibit sterility and/or embryonic lethality at all temperatures [84, 85]. Homozygous males are sterile at 25 °C, but produce a significant number of cross progeny at 20 °C [25, 86]. Taking advantage of the fertility of homozygous males raised at 20 °C, Conine et al. [25] demonstrated that heterozygous progeny derived from mating with homozygous males showed a progressive transgenerational sterility at 25 °C, while heterozygous progeny derived from mating with heterozygous males showed normal fertility [25, 84]. This progressive sterility of heterozygous hermaphrodites was rescued by mating with wildtype males, indicating that hermaphrodites produced fertile oocytes but had defective sperm. *Sup-46*-dependent transgenerational sterility was not rescued by mating with normal males. This finding is consistent with the observed failure of oocyte formation in *sup-46* sterile hermaphrodites, and implies that a distinct mechanism underlies the transgenerational sterility in mated *csr-1* and *alg-3,4* heterozygous mutants, compared with the *sup-46* homozygous mutant. We also determined that sterility of unmated *sup-46* mutant hermaphrodites treated with *csr-1(RNAi)* was much more severe than wildtype worms treated with *csr-1(RNAi)* or *sup-46* mutants treated with control RNAi [*empty vector(RNAi)*] (Fig. 6c). These results further support the conclusion that CSR-1 and SUP-46 have additive and distinct modes of action in preventing transgenerational sterility.

SUP-46 depletion resulted in dissipated P granules in the male germline, and P granules contain small RNA molecules that might transmit epigenetic information, analogous to what has been described in the sperm chromatoid body in mammals [87]. To test whether P granule disruption in the male germline was responsible for the mating-dependent transgenerational sterility, we examined the effect of mating on sterility in *glh-1(ok439)*, which, like the *sup-46* mutant, had dissipated germline P granules at 20 °C. The *glh-1* mutant had a slow, modest (2.5-fold) increase in sterility, from a baseline of 8% (Fig. 6b, inset). In contrast, the *sup-46* mutant exhibited an exponential, 60-fold increase in sterility from 1% at generation 1 to 62% at generation 18 (Fig. 6b). Sterile *sup-46* mutant worms were almost exclusively of the ventral white patch (no oocyte) phenotype, while the *glh-1* mutants primarily laid eggs that failed to hatch. These results indicate that, while *sup-46* mutant males have dissipated P granules, this probably does not account for the mating-dependent transgenerational sterility.

## Discussion

Epigenetic changes in germline cells can provide gametes with a heritable history of environment that, ultimately, contributes to species survival. Multiple classes of molecules are implicated in transmission of epigenetic information, including histones and non-coding RNA. In this study we identified a new molecule, SUP-46 RNA-binding protein, that is required for germline immortality in *C. elegans* hermaphrodites. Sterility developed in *sup-46* mutants at room temperature within just a few generations, indicating a crucial role for SUP-46 in maintaining germline immortality at physiological conditions. Perhaps the most intriguing and unexpected finding was that sterility in the mutant occurred when oocytes were fertilized by male sperm, but not hermaphrodite sperm. This raises the exciting possibility that identifying molecular dimorphisms in male and hermaphrodite sperm will reveal novel regulators of epigenetic inheritance.

### SUP-46, non-coding RNA, and epigenetic inheritance

At fertilization, the sperm transfers DNA with its associated chromatin, as well as nucleoplasm and cytoplasm*. C. elegans* sperm cytoplasm has multiple components, including mitochondria, membranous organelles, mRNA, and ncRNAs bound to AGO and other RNA-binding proteins [12, 88]. Despite being much smaller than the oocyte, the *C. elegans* sperm contributes 50% or more of the zygotic small ncRNA, including siRNA (22G and 26G RNAs), piRNA, and miRNA [12]. Loss of sperm-provided 22G siRNA results in progressive transgenerational sterility in *alg-3,4* or *csr-1* heterozygous hermaphrodite progeny raised at 25 °C [25]. Of the genes identified as targeted by sperm-provided 22G RNA [12], only 5% (9/195) were increased (*P* = 0.1), and 4% (7/195) decreased (*P* = 0.44), in the *sup-46* mutant. Moreover, we found that co-depletion of *sup-46* and *csr-1* resulted in a large synthetic increase in sterility within two generations, consistent with their acting in parallel pathways. In addition, while transgenerational sterility of *csr-1* or *alg-3,4* heterozygote mutants is rescued by mating with wildtype males [25], *sup-46* mutants exhibited oogenesis defects that were not rescued by mating with wildtype males. Taken together, these data support the conclusion that SUP-46 and CSR-1 are both required to prevent germline mortality, but act in mechanistically distinct ways.

A role for miRNA in epigenetic inheritance in *C. elegans* has not been reported. However, in mice, sperm miRNA transmit epigenetic information over multiple generations [11, 89]. HNRNPM and MYEF2 bound multiple proteins of the DROSHA microprocessor complex and SUP-46 interacts with DCR-1 [90], which is required for miRNA processing. These findings point to a possible role for SUP-46 in generating miRNA that might act as carriers of epigenetic information in male sperm. *C. elegans* has more than 400 miRNA [91]. Thirteen miRNA are enriched in the hermaphrodite germline, including the *miR-35-41* miRNA cluster [91]. In a *miR-35-41* mutant, 553 genes were upregulated and 1080 downregulated [92]. Of the upregulated genes, in the *sup-46* mutant, 6% (31/553) were increased (*P* = 0.08) and 5% (29/553) decreased (*P* = 0.11). Of those downregulated in the *miR-35-41* mutant, 6% (67/1080) were increased (*P* = 0.1) but only 2% (17/1080) (*P* = 9 × 10^–7^) were decreased, representing a 2.6-fold under-enrichment. It is not known whether these same miRNA are carried in sperm, but the modest under-enrichment of *mir-35-41* mutant downregulated genes in the *sup-46* mutant might suggest a role for altered delivery of *mir-35-41* miRNA in the paternally-induced transgenerational sterility in *sup-46* mutants. A role for other miRNA remains to be examined.

Like small ncRNA, lncRNA have been widely implicated in epigenetic regulation [93]. HNRNPM and MYEF2 have been identified as components of at least two lncRNA compartments, namely the X chromosome inactivation center and the paraspeckle [45, 48, 49], and we confirmed interaction with multiple XIST and paraspeckle proteins. In the *sup-46* mutant, we did not see obvious defects in X chromosome chromatin marks, and only very modest changes in expression of X chromosome genes (3% increased, 2% decreased in the *sup-46* mutant), indicating that SUP-46 does not play a major role in X chromosome inactivation in *C. elegans*. It is unknown if SUP-46 localizes to paraspeckles. However, its distribution overlaps with *C. elegans* NONO-1 in nucleoli and mammalian NONO localizes to nucleoli and paraspeckles [17, 94]. In mammals, NONO-family proteins in the paraspeckle act as epigenetic regulators of transcription, post-transcriptional processing, and localization of coding and ncRNA [47]. Future experiments examining a role for NONO-1 and other putative paraspeckle components in transgenerational sterility will help to determine if SUP-46-dependent lncRNA are important for epigenetic regulation and germline immortality in *C. elegans.*

In addition to ncRNA, sperm transfer histone proteins that can act as epigenetic regulators by altering gene transcription. Failure to demethylate histone H3K4 in *spr-5* mutants causes transgenerational sterility in selfing hermaphrodites, beginning by generation 15 [18]. Similar to the *sup-46* mutant, the *spr-5* mutant has defects in both oogenesis and spermatogenesis, and increased abundance of many spermatogenesis transcripts. A comparison of spermatocyte H3K4me2 and H3K27me2me3 abundance did not show differences in male *sup-46* mutants at generation 1, compared with generation 17. Selfing *sup-46* mutant hermaphrodites also did not develop progressive sterility, at least up to generation 25. These findings may suggest that sperm-mediated delivery of hypermethylated histone H3K4 at spermatogenesis genes does not underlie *sup-46* mutant transgenerational sterility. However, immunofluorescence of condensed sperm is not of sufficient resolution to detect differences in histone methylation at specific genes. Accordingly, additional experiments, such as H3K4me2 chromosome immunoprecipitation, would be required to determine if histone changes at one or more loci contribute to the transgenerational sterility phenotype. Moreover, a role for other histone modifications remains to be examined.

### SUP-46 regulates gametogenesis

Our RNASeq data showed that depleting SUP-46 increases spermatogenesis transcripts and, to a lesser extent, decreases oogenesis transcripts. In *C. elegans*, 3’UTRs regulate expression of oocyte genes, whereas spermatogenesis genes are regulated by promoters acting at the level of transcription [95]. Imaging of the male germline demonstrated that SUP-46 was strongly chromatin-associated until just before meiosis I anaphase, at which point it abruptly accumulated in cytoplasmic granules before eventual loss in the residual body. Loss of nuclear SUP-46 correlates temporally with transcription of sperm-specific genes [96], and the *sup-46* mutant had a partially penetrant phenotype of premature spermatogenesis in the middle part of the germline. Taken together, our data suggest that removal of nuclear SUP-46 may be necessary for transcription of spermatogenesis genes and completion of meiosis. We determined that the P granule mutant *glh-1(ok439)* also had premature sperm, phenocopying the *sup-46* mutant, and that SUP-46 depletion disrupted P granules. As such, disruption of P granules in the *sup-46* mutant may partially explain the ectopic sperm and increased spermatogenesis transcripts. However, P granule depletion causes increased abundance of transcripts encoding HER-1 and FOG-3, which promote sperm fate [76], which was not seen in the *sup-46* mutant. Moreover, the *sup-46* mutant had decreased oogenesis transcripts, which was not seen following P granule depletion. Therefore, SUP-46 and P granules each have roles in gametogenesis that are independent of one another.

## Conclusions

In this study, we identified the *C. elegans* SUP-46 HNRNPM family RNA-binding protein. We provided evidence that SUP-46 and its human homologs, MYEF2 and HNRNPM, localize to multiple RNA granules, including stress granules. SUP-46 was crucial for stress resistance, and was also needed to spatially restrict spermatogenesis to the proximal part of the germline. Intriguingly, SUP-46 prevented paternally-mediated transgenerational sterility. Experiments in mice and humans show that environmental stress can result in paternally-mediated epigenetic changes that result in transgenerational morbidity. With humans living longer than ever, a key goal in medicine is to understand how environmental exposure leads to epigenetic changes that alter outcomes in chronic diseases of aging. Future studies on the mechanisms underlying SUP-46, HNRNPM, and MYEF2 epigenetic inheritance should prove to be highly informative.

## Methods

### Cloning and nematode strains

Nematodes were cultured on NGM plates seeded with OP50 bacteria using standard techniques [97] and maintained at 20 °C, unless otherwise noted. DNA constructs were made by fosmid recombineering [98] and/or standard molecular cloning techniques, and PCR-derived fragments were sequence-verified. For plasmid pAK182-3, which encodes the ΔMYEF2 motif::FLAG::GFP, a single base-pair deletion was found in the non-coding region (exon3). Transgenic lines were produced by biolistic transformation [99] or by MosSCI [100, 101] and protein expression of GFP-tagged deletion mutants was verified by immunoblotting (for details please see Additional file 9). Names of plasmids used to generate transgenic animals, and worm strain names and genotypes are outlined in Additional file 1: Table S7, and annotated sequence files of the plasmids are available on request.

### SNP mapping, sequencing of mutants, and RNAi

Detailed SNP mapping was performed by crossing XA789 [*gna-2(qa705) unc-55(e1170) sup-46(qa710) unc-101(m1)I*] with the Hawaiian strain CB4856, and isolating 182 homozygous F3 fertile recombinant lines that showed an Unc-55, non-Unc-101 phenotype. The lines were queried for Hawaiian versus Bristol snipSNP status by PCR and restriction digest to place the suppressor to the left of snp_T20F10.1. *Sup-46(qa707)* DNA was prepared as outlined in Additional file 9 for whole genome sequencing by the Michael Smith Genome Sciences Centre (Vancouver, Canada). Tentative identification of the mutation was kindly provided by Don Moerman and Stephane Flibotte (*C. elegans* Knockout Facility, Vancouver, Canada) using the SNP mapping and whole genome sequencing data. Mutations were identified by PCR and DNA sequencing in the other three mutant alleles *(qa708-710)*. Two independent approaches were used to show that depletion of C25A1.4 was responsible for the *sup-46* mutant phenotype. Firstly, by *C25A1.4(RNAi)* to test for phenocopy of *sup-46* mutant suppression of *gna-2(qa705)* embryonic lethality and, secondly, by restoration of embryonic lethality in *gna-2(qa705) sup-46(qa708)* by SUP-46::FLAG::GFP. Experimental details are provided in Additional file 9.

### Identifying SUP-46 homologs and the MYEF2 motif

NCBI BLAST [102] of full-length SUP-46 was used to identify SUP-46 homologs in human and *Drosophila*. Putative protein domains were found using Expasy PROSITE [103], and NCBI BLAST of the LC domain identified a short (24 AA) region conserved in nematodes. Subsequent BLAST of the 24 AA region uncovered a 20 AA motif that is highly conserved (70% identical in worm and mammals) in HNRNPM/MYEF2 family proteins, but not in other HNRNP families. A sequence logo for this motif (designated the MYEF2 motif) was created using WebLogo3.4 (http://weblogo.threeplusone.com; [104, 105]) and the conserved 20 AA LC domain (AA 276-295 in *C. elegans*) from 61 species, including 4 *Caenorhabditis* nematodes, 4 non-*Caenorhabditis* nematodes, 4 insects, 2 molluscs, 2 fish, 4 reptiles, 5 birds, and 36 mammals (Additional file 1: Table S8).

### RNASeq and overlap with gametogenesis and small ncRNA transcripts

RNASeq analysis was performed on three biological replicates of young adult N2 (wildtype) or *sup-46(qa710)* worms. Details of sample preparation are provided in Additional file 9. Sequencing was done on an Illumina HiSeq2000 platform with TruSeq SBS v3, and the real-time base call (.bcl) files were converted to fastq files using CASAVA 1.8.2 (on CentOS6.0 data storage and computation Linux servers). Sequences in fastq format were aligned to WS220 version of WormBase (http://www.wormbase.org) using RNASeq unified mapper version 2.0.1 [106]. The results were processed with custom Perl scripts to generate gene counts for all genes in WS220 in each of the triplicates of N2 and *sup-46(qa710).* The DESeq [107] expression analysis package in R/BioConductor was utilized to identify the relative expression difference of genes in N2 and *sup-46(qa710)*, and genes that had a reported *P* ≤ 0.05 were deemed differentially expressed. To examine alternative splicing, transcripts in the WS220 version of WormBase were classified into different types of alternative splicing events using two different methodologies, namely DEDB (http://proline.bic.nus.edu.sg/dedb/methodology.html) and SpliceTrap (http://rulai.cshl.edu/splicetrap/). Information on the classes of alternative splicing events and the classification process are available on the websites corresponding to the methods. Splice junctions were further classified into known and novel splice junctions using a custom Perl script, which takes as input genome coordinates of all annotated exons and all predicted splice junctions. Finally, previously mapped reads were compared to splice event locations and, for each of these events, the number of reads mapping to every junction associated with the alternative splicing event were counted in both wildtype and *sup-46(qa710).* Events were excluded from statistical analysis if there were no reads mapping to them in either genotype. The events passing the filters were tested for statistical significance using Fisher’s exact test and alternative splicing events that had *P* < 0.05 were considered to be significant.

RNASeq data showing significant differences between N2 and *sup-46(qa710)* were compared with RNA datasets from [74–76]. Spermatogenesis and oogenesis transcript sets were defined as those transcripts identified as sperm or oocyte enriched by both Reinke et al. [75] and Ortiz et al. [74]. Venn diagrams were generated using the online tool Venny [108]. Sperm-provided 22G RNA were retrieved from Stoeckius et al. [12], and *mir35-41* miRNA, from Massirer et al. [92]. Significance of overlap between data sets was calculated using the online hypergeometric *P* value calculator (http://systems.crump.ucla.edu/hypergeometric/index.php) provided by T. Graeber (Los Angeles, CA) to determine the cumulative distribution function of the hypergeometric distribution, assuming 20,466 protein-coding genes (WormBase Release WS244).

### Microscopy and immunohistochemistry

Imaging was performed at room temperature (20–22 °C) using a LEICA DMRA2 microscope (Fig. 6a) or by confocal microscopy (remainder of imaging) using a Leica DMLSFA or a NIKON Ti-E inverted confocal microscope. For each confocal image within a Figure panel, laser intensity and gain were the same, unless otherwise noted. Details of microscope settings are provided in Additional file 9. For Additional file 6, GNA-1 protein in *sup-46* mutant and wildtype gonads was quantified by measuring average pixel intensity in a frontal plane section positioned through the middle of the gonad rachis. For Additional file 8, histone H3K4me2 and H3K27me2me3 were quantified by measuring average pixel intensity in five spermatocytes for each of five gonads. Average pixel intensities were compared by ANOVA, with differences between genotypes or generations tested by Bonferroni’s Multiple Comparison Test (GraphPad Prism 4.02).

Live imaging of adult worms was performed by anaesthetizing worms in 10 μM levamisole on 2% agarose pads. For imaging of embryos, hermaphrodites were transferred to Buffer A (150 mM KCl, 5 mM HEPES, pH 7.5) on a coverslip, and embryos were released by cutting the hermaphrodite at the vulva, with the coverslip then inverted onto a 2% agarose pad. To generate Movie 3, two zygotes were imaged for 33 minutes beginning just after pronuclear decondensation to the four-cell embryonic stage. Forty-five images were collected manually (approximately every 30–90 seconds), and rendered into a movie using Photoshop CS5, with a 0.3 second delay between frames.

For fixed imaging, worms were washed in Buffer A or Buffer B (50 mM KCl, 100 mM NaCl, 2 mM EDTA, 500 μM EGTA, 25 mM HEPES, pH 7.4, 0.1% Tween-20; adapted from [109]) and transferred to slides coated with 0.1% (w/v) polylysine (Sigma). Gonads were released by cutting just behind the pharynx, and embryos by cutting at the vulva. For worms dissected in Buffer B, paraformaldehyde was added to a final concentration of 3.7%, which provided superior fixation of male gonads. Dissected samples were covered with a glass coverslip, flash-frozen in dry ice, and the cover slip pried off quickly (freeze-fractured). Samples dissected in Buffer A were fixed in –20 °C methanol for 8 minutes (except CGH-1 antibody experiments, which used a 1 minute fixation time). All slides were washed subsequently in PBS with 0.5% Tween-20, and blocked in PBS with 10% goat serum plus 1% BSA for 1 hour prior to incubation with antibodies, as outlined in Additional file 9.

### Heat stress, fecundity, and survival

To measure fecundity following chronic temperature stress, *sup-46(+)*, *sup-46(qa707)*, *sup-46(qa710)*, and *sup-46(qa708)* were maintained at the indicated temperatures (12 °C, 16 °C, 20 °C, 26 °C). Fecundity was also measured in *sup-46(+)* and *sup-46(qa708)* maintained at 26 °C and fed control *empty vector(RNAi)* or *gna-1(RNAi)*. Single eggs were transferred to individual plates (n = 11–35), and hatchling number/hermaphrodite was quantified following daily transfer of the hermaphrodite to a fresh plate until no new eggs were laid. Hatchling numbers were compared by ANOVA, with differences between genotypes tested by Bonferroni’s Multiple Comparison Test (GraphPad Prism 4.02).

To test acute heat stress sensitivity, strains were maintained at 20 °C and comma staged embryos were identified under a dissecting microscope and transferred to individual NGM plates seeded with OP50 bacteria (20 embryos/plate (replicate); 10–14 replicates/genotype). Plates were sealed with Parafilm and floated in a 36 °C water bath for 15–90 minutes. Preliminary experiments using a low mass thermocouple and circuit board from a Koolatron PC3 incubator indicated that the time for the OP50 lawn/NGM agar surface to reach 36 °C in the water bath was less than 5 minutes. Following heat stress, plates were floated in a 20 °C water bath for 5 to 10 minutes and then transferred to a 20 °C dry incubator overnight. The next day, survival (percentage of embryos on the plate that hatched) was quantified, and plates were kept for 3 to 4 days to ensure no further hatching. Survival was normalized relative to 0 time heat shock control, and fit to a sigmoidal dose-response curve, with F tests used to determine significant differences between genotypes (GraphPad Prism 4.02).

Imaging of SG formation and dissolution was performed following incubation in the 36 °C water bath, with recovery for the indicated time at 20 °C prior to dissection and mounting for viewing. The earliest recovery time point imaged (~5 minutes) represents the time required to dissect and prepare the embryo for microscopy. All other imaging of SG was performed following 3 hours heat stress in a 36 °C dry incubator. Preliminary experiments demonstrated robust formation of SG by 60–90 minutes in the dry incubator, with negligible recovery for at least 60 minutes, facilitating dissection and processing of embryos for immunohistochemistry and imaging.

### Transgenerational sterility assay

Experiments were conducted at 20 °C. Prior to the assay, worm strains were maintained on NGM plates containing OP50 bacteria for at least three generations without starving. To determine baseline sterility at Generation 0 (Gen0) for the “selfed” *sup-46(qa710)* treatment, L4 hermaphrodites were transferred to each of seven NGM plates containing OP50 bacteria. Six “test” plates were used for the sterility assay, while the seventh plate was used to generate worms for the subsequent generation (Gen1). The next day, worms on the test plates were examined under a dissecting microscope, and those with refractile, well-formed oval embryos in utero were counted, removed, and recorded as “fertile.” All other worms were cloned onto individual plates and monitored for at least 3 days to determine if they produced any live progeny (“fertile”) or failed to produce any live progeny (“sterile”). The assay was continued up to Gen25. For the sibling mated (“sib-mated”) *sup-46(qa710)* and *glh-1(ok439)* treatments, baseline sterility at Gen0 was determined as outlined above. In addition, 10–20 spontaneous males were collected from 4 to 6 100 mm × 15 mm NGM plates, and crossed with 10–15 L4 hermaphrodites. Following overnight mating, hermaphrodites were cloned onto individual plates and monitored for the presence of abundant male progeny (indicating successful mating). From plates with abundant males, a total of 60 males were transferred to a single plate containing 30 L4 hermaphrodites (from unmated plates), taking care to take the same number of males from each of the successfully mated plates. Following overnight mating, hermaphrodites were cloned onto individual plates and monitored for the presence of male progeny. From plates with male progeny, a total of 60 males and 330 L4 hermaphrodites were collected, taking care to collect the same number of males and hermaphrodites from each plate. One plate, containing the 60 males and 30 L4 hermaphrodites was used to set up mating of the siblings to generate progeny for the next generation. The remaining L4 hermaphrodites were transferred to six test plates (50 worms/plate), which were assayed for sterility (Gen1) as described above. The sib-mated *sup-46(qa710)* assay was continued until Gen 18, at which point there were insufficient progeny to set up mating for the next generation of progeny.

To test for a synthetic effect of depleting SUP-46 and CSR-1, 10 L3 P_o_ (parent) hermaphrodites (wildtype or Gen1 *sup-46(qa710)*) were transferred to each of five control *(mtv(RNAi))* or *csr-1(RNAi)* plates. From each plate, 50 L4 F_1_ progeny were subsequently removed to NGM plates seeded with OP50 bacteria and examined for sterility, as described above. Feeding *csr-1(RNAi)* over multiple generations is embryonic lethal, so removal of the L4 F_1_ progeny to OP50 plates allowed sufficient L4 F_2_ progeny (P_o_ exposed to *csr-1(RNAi)*) to be generated and transferred to fresh plates (*mtv(RNAi) or csr-1(RNAi)*) for assay of sterility at Gen2. The percentage of sterile worms were compared by ANOVA, with differences tested by Bonferroni’s multiple comparison test (GraphPad Prism 4.02).

### Proximity biotinylation coupled to mass spectrometry (BioID-MS)

Stable human (taxid:9606) cells (Flp-In T-REx 293 cells) were generated expressing the human SUP-46 homologs (HNRNPM and MYEF2) fused to FLAG-BirA. Expression of HNRNPM::FLAG-BirA and MYEF2::FLAG-BirA was induced by 1 μg/mL tetracycline, and media was supplemented with 50 μM biotin for protein labeling. Cells were grown, lysed in modified RIPA buffer, affinity purified using streptavidin-sepharose beads, and digested with trypsin prior to mass spectrometry. Details of transfection, protein preparation, mass spectrometry, data analysis, and Significant Analysis of Interactome are provided in Additional file 9.

## Additional files

Additional file 1: Xcel file containing Tables S1–S8. **Table S1** Partial rescue of *gna-2(qa705)* maternal effect embryonic lethality by *sup-46(RNAi).* **Table S2** HNRNPM and MYEF2 SAINT table. **Table S3** HNRNPM and MYEF2 proximal proteins in RNA granules and RNA-associated complexes. See also Fig. 2. **Table S4** Proteins of the nucleolus (cellular compartment GO:0005730; 862 proteins) associated with HNRNPM and MYEF2. **Table S5** RNASeq data of transcript abundance in wildtype and *sup-46(qa710)*. **Table S6** Transcripts showing splicing differences between wildtype and *sup-46(qa710)*. **Table S7** Worm strains generated in this study. **Table S8** Sequences used to generate the MYEF2 sequence logo. (XLSX 1939 kb)

Additional file 2: SUP-46 is absent from nucleoli in the loop region of the hermaphrodite gonad. Movie is a confocal z series through the gonad of a live hermaphrodite expressing SUP-46::FLAG::GFP, and corresponds to Fig. 3c, left-hand image. SUP-46 is present diffusely in the nucleus, as well as associated with chromatin, but is absent from nucleoli (outlined by hatched circle). Gonad is outlined by a hatched line. Compare with Additional file 3 which shows accumulation in nucleoli of nuclei in the oogenesis region of the germline. (MOV 152 kb)

Additional file 3: SUP-46 accumulates in nucleoli in the oogenesis region of the hermaphrodite gonad. Movie is a confocal z series through the gonad of a live hermaphrodite expressing SUP-46::FLAG::GFP, and corresponds to Fig. 3c, right-hand image. SUP-46 is present diffusely in the nucleus, as well as associated with chromatin, and is also present in nucleoli (outlined by hatched circle). Gonad is outlined by a hatched line. Compare with Additional file 2, which shows absence of SUP-46 in nucleoli of nuclei in the loop region of the germline. (MOV 211 kb)

Additional file 4: Two early embryos show SUP-46 shuttles between the nucleus and the cytoplasm during cell division. SUP-46 is depleted from the nucleus just before cell division and re-accumulates in the nucleus after cell division. SUP-46 can also be seen in P granules. Movie spans 33 minutes, from nuclear decondensation in the newly fertilized zygote to the 4-cell embryo. Strain expresses SUP-46::FLAG::GFP. (MOV 8628 kb)

Additional file 5: SUP-46 localization to stress granules may depend on the MYEF2 motif. (A) TIAR-2 and CGH-1 colocalize in heat stress-induced granules in embryos. Strain expresses LAP::TIAR-2. CGH-1 was detected with an anti-CGH-1 antibody, and WGA lectin marks the eggshell. (B) Following heat stress SUP-46 colocalizes with TIAR-2 in granules, confirming SG localization of SUP-46. In the bottom row overlay image, the box in the upper right corner shows enlarged SG. Strain expresses LAP::TIAR-2 and SUP-46::FLAG::mCherry. (C) GFP-tagged SUP-46 accumulates in SG (top row), which is not seen with GFP alone (second row) or with GFP-tagged tubulin (cytoplasmic protein), histone H2B (nuclear protein), or EGG-1 (membrane protein) (rows 3–5). Laser intensity and gain is identical within rows. (D) Immunoblot of strains deleted for different regions of the SUP-46 protein and probed with anti-GFP antibody. γ-tubulin was used as a loading control, and molecular weight markers (kD) are indicated on the left side of the blot. Arrowheads on the right-hand side of bands indicate the GFP-tagged SUP-46 deletion proteins. (E) Localization of SUP-46 to SG may require the MYEF2 motif. Cartoons on the left indicate regions deleted (jointed lines) in the GFP-tagged proteins. The relative GFP accumulation in SG is indicated from none (“-”) to highly accumulated (“++++”). All strains were generated by MosSCI, and images are single confocal sections of live 2–4 cell embryos, with laser intensity and gain identical within each row. (A–C, E) Scale bars represent 15 μm. (PDF 1232 kb)

Additional file 6: SUP-46 and GNA-1. (A) GNA-1 protein is increased in *sup-46* mutants. Images show examples of *gna-1::gfp-*tagged wildtype and *sup-46* mutants, with the gonad region used to quantify GNA-1::GFP traced by the white line. The graph on the right provides quantification. Error bars represent SD (n = 15–16). * significantly different from wildtype [*sup-46(+)*]; *P* < 0.05. Images are single confocal optical sections of live young adult hermaphrodites. Scale bars represent 15 μm. (B) SUP-46 and GNA-1 are each required for maximum fecundity at elevated temperatures (26 °C). Data points represent brood counts of individual hermaphrodites. Hatched line and error bars are the mean ± SD (n = 18–40). * significantly different from control [*empty vector(RNAi)*] for that genotype, *P* < 0.05. (PDF 181 kb)

Additional file 7: SUP-46 regulates the abundance of two rare *tdp-1* splice junctions and SUP-46 and TDP-1 both localize to somatic nuclei. (A) Cartoon shows that, in *sup-46(qa710)*, the rare junction between exons 3 and 5 of *tdp-1* is decreased, and the rare junction between exons 5 and 6 is increased. Cartoon shows splice junctions for *tdp-1*. * For each junction, the number on top (bold font) is the relative abundance of that splice junction in *sup-46(qa710)* compared to the relative abundance in wildtype, as identified by RNASeq. Relative abundance of splice junctions was calculated by determining the number of a particular *tdp-1* splice junction, as a percent of the total number of *tdp-1* junctions detected for that genotype [*sup-46(qa710)* or wildtype]. The numbers below (grey font, in parentheses) are the total number of a particular junction detected in *sup-46(qa710)* (first number in pair) or wildtype (second number in pair). Splice junctions are indicated with jointed lines between exons. Rare junctions (2% or fewer of total) are indicated in red, and were altered in relative abundance in *sup-46(qa710)*. Regions encoding the NLS and RRM domain motifs are indicated. (B) Distribution of SUP-46 and TDP-1 overlaps in somatic nuclei. Examples indicated are head neurons (blue arrow) and intestine (white arrow). Image is a composite of a live young adult hermaphrodite with the two overlapping single confocal optical sections stitched together. Scale bar represents 15 μm. (PDF 940 kb)

Additional file 8: Spermatocyte histone H3K4me2 and histone H3K27me2me3 appear similar between Generation 1 and Generation 17 males. Quantification of anti-H3K4me2 and anti-H3K27me2me3 in spermatocyte nuclei shows equivalent signal intensity of active (H3K4me2) and inactive (H3K27me2me3) histone marks in generation 1 (Gen 1) and Gen 17 male *sup-46* mutants. Germlines were dissected, fixed, and stained with antibodies and PicoGreen (DNA). Scale bars represent 15 μm. Images are of a single confocal optical plane, with laser intensity and gain identical between samples. To ensure detection of the fluorescence signal was equivalent in spermatocytes that were on different focal planes, the histone signals were normalized to PicoGreen (DNA) intensity from the same spermatocyte. Hatched line and error bars are the mean ± SD (n = 5). ns, not significantly different (*P* > 05). (PDF 354 kb)

Additional file 9: Additional methods. (DOCX 41 kb)
