# Supplementary figures and images for: C. elegans SUP-46, an HNRNPM family RNA-binding protein that prevents paternally-mediated epigenetic sterility
Source: BMC Biol. 2017 Jul 17;15:61. doi: 10.1186/s12915-017-0398-y (PMC5513350; doi:10.1186/s12915-017-0398-y)

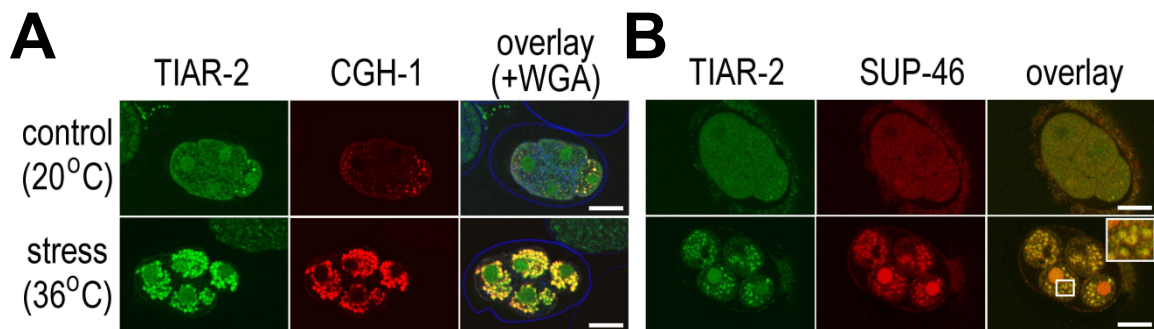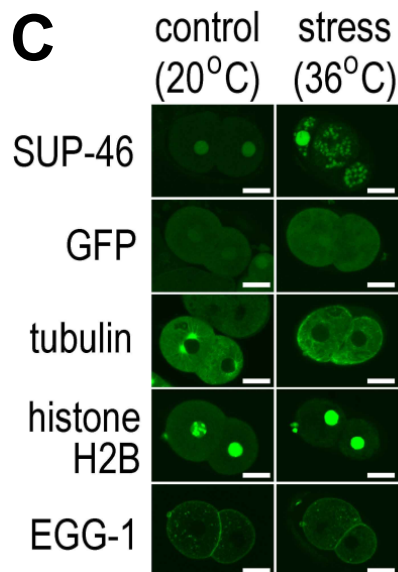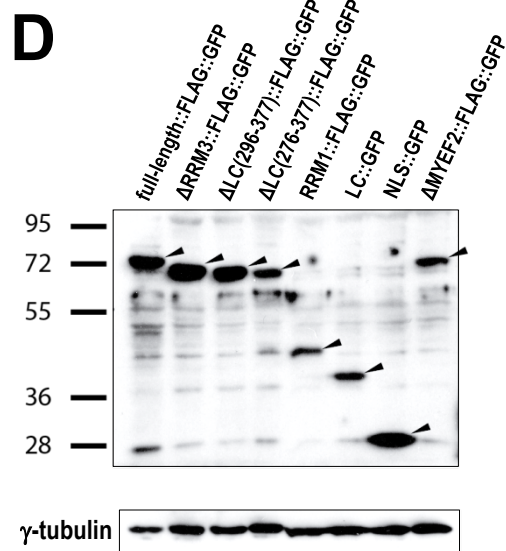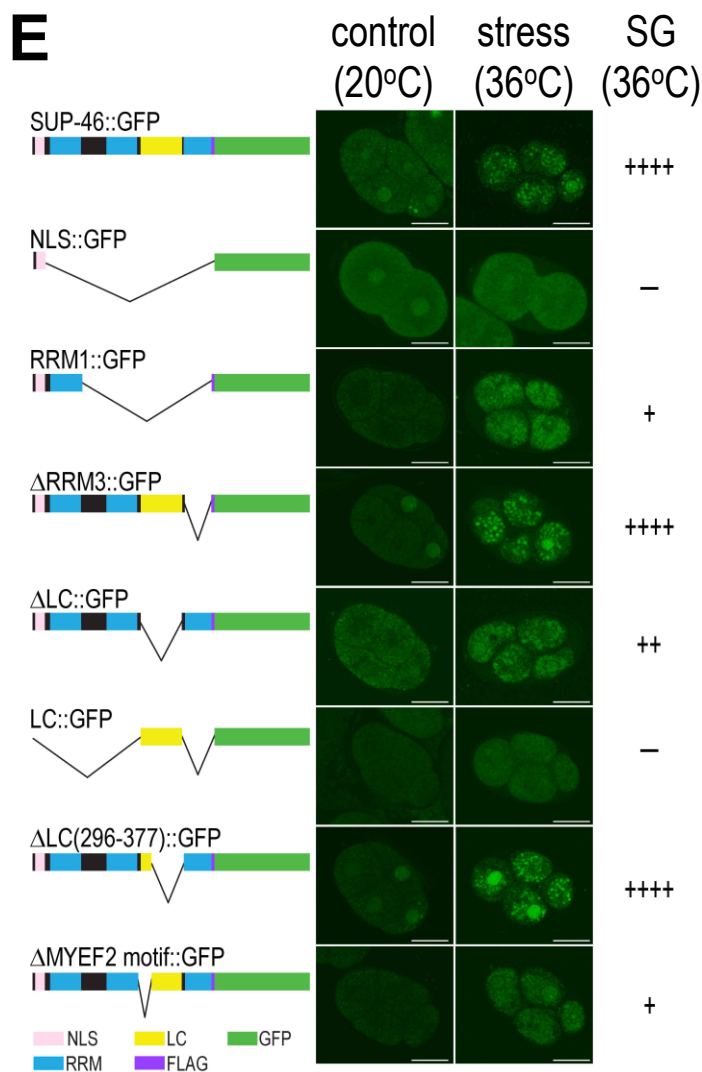

Supplement: Supplementary file 5 — SUP-46 localization to stress granules may depend on the MYEF2 motif. (A) TIAR-2 and CGH-1 colocalize in heat stress-induced granules in embryos. Strain expresses LAP::TIAR-2. CGH-1 was detected with an anti-CGH-1 antibody, and WGA lectin marks the eggshell. (B) Following heat stress SUP-46 colocalizes with TIAR-2 in granules, confirming SG localization of SUP-46. In the bottom row overlay image, the box in the upper right corner shows enlarged SG. Strain expresses LAP::TIAR-2 and SUP-46::FLAG::mCherry. (C) GFP-tagged SUP-46 accumulates in SG (top row), which is not seen with GFP alone (second row) or with GFP-tagged tubulin (cytoplasmic protein), histone H2B (nuclear protein), or EGG-1 (membrane protein) (rows 3–5). Laser intensity and gain is identical within rows. (D) Immunoblot of strains deleted for different regions of the SUP-46 protein and probed with anti-GFP antibody. γ-tubulin was used as a loading control, and molecular weight markers (kD) are indicated on the left side of the blot. Arrowheads on the right-hand side of bands indicate the GFP-tagged SUP-46 deletion proteins. (E) Localization of SUP-46 to SG may require the MYEF2 motif. Cartoons on the left indicate regions deleted (jointed lines) in the GFP-tagged proteins. The relative GFP accumulation in SG is indicated from none (“-”) to highly accumulated (“++++”). All strains were generated by MosSCI, and images are single confocal sections of live 2–4 cell embryos, with laser intensity and gain identical within each row. (A–C, E) Scale bars represent 15 μm. (PDF 1232 kb) [file 12915_2017_398_MOESM5_ESM.pdf]

**A**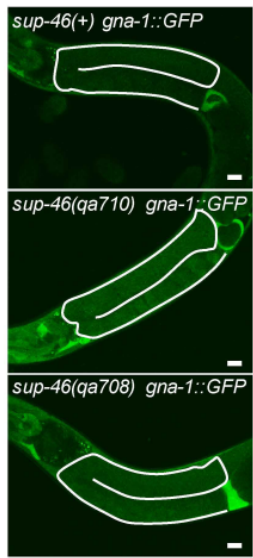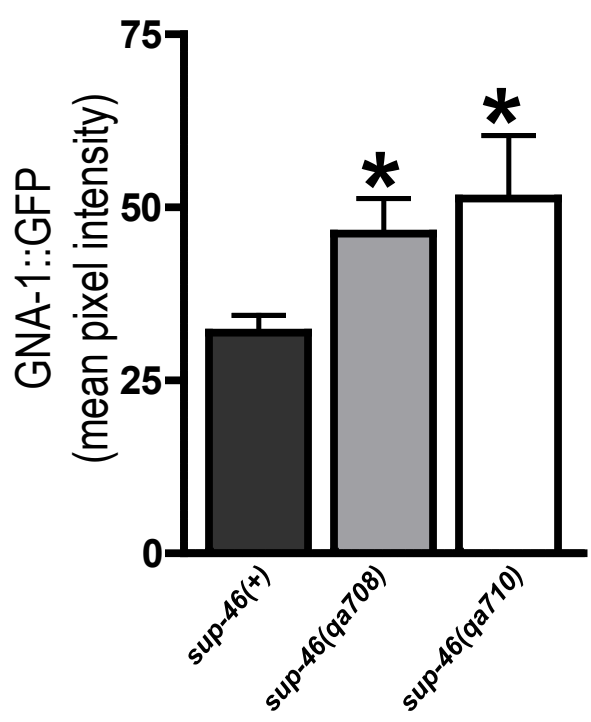**B**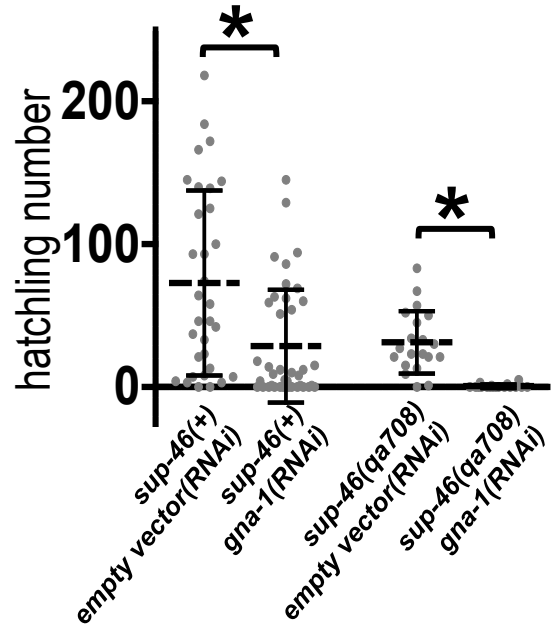

Supplement: Supplementary file 6 — SUP-46 and GNA-1. (A) GNA-1 protein is increased in sup-46 mutants. Images show examples of gna-1::gfp-tagged wildtype and sup-46 mutants, with the gonad region used to quantify GNA-1::GFP traced by the white line. The graph on the right provides quantification. Error bars represent SD (n = 15–16). * significantly different from wildtype [sup-46(+)]; P < 0.05. Images are single confocal optical sections of live young adult hermaphrodites. Scale bars represent 15 μm. (B) SUP-46 and GNA-1 are each required for maximum fecundity at elevated temperatures (26 °C). Data points represent brood counts of individual hermaphrodites. Hatched line and error bars are the mean ± SD (n = 18–40). * significantly different from control [empty vector(RNAi)] for that genotype, P < 0.05. (PDF 181 kb) [file 12915_2017_398_MOESM6_ESM.pdf]

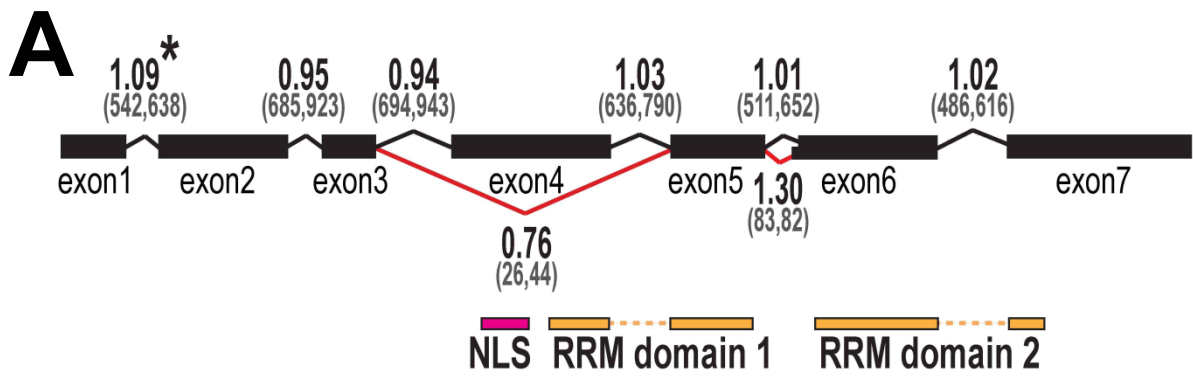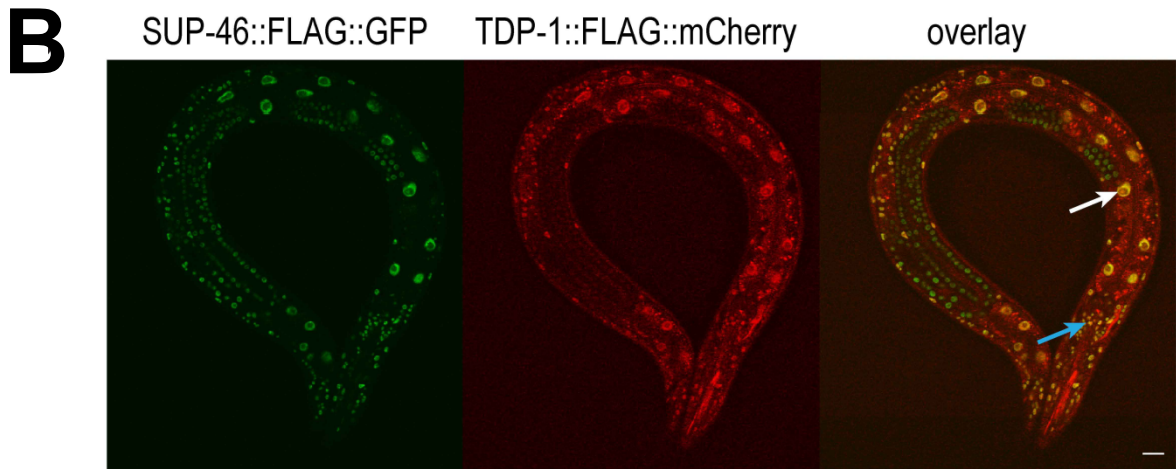

Supplement: Supplementary file 7 — SUP-46 regulates the abundance of two rare tdp-1 splice junctions and SUP-46 and TDP-1 both localize to somatic nuclei. (A) Cartoon shows that, in sup-46(qa710), the rare junction between exons 3 and 5 of tdp-1 is decreased, and the rare junction between exons 5 and 6 is increased. Cartoon shows splice junctions for tdp-1. * For each junction, the number on top (bold font) is the relative abundance of that splice junction in sup-46(qa710) compared to the relative abundance in wildtype, as identified by RNASeq. Relative abundance of splice junctions was calculated by determining the number of a particular tdp-1 splice junction, as a percent of the total number of tdp-1 junctions detected for that genotype [sup-46(qa710) or wildtype]. The numbers below (grey font, in parentheses) are the total number of a particular junction detected in sup-46(qa710) (first number in pair) or wildtype (second number in pair). Splice junctions are indicated with jointed lines between exons. Rare junctions (2% or fewer of total) are indicated in red, and were altered in relative abundance in sup-46(qa710). Regions encoding the NLS and RRM domain motifs are indicated. (B) Distribution of SUP-46 and TDP-1 overlaps in somatic nuclei. Examples indicated are head neurons (blue arrow) and intestine (white arrow). Image is a composite of a live young adult hermaphrodite with the two overlapping single confocal optical sections stitched together. Scale bar represents 15 μm. (PDF 940 kb) [file 12915_2017_398_MOESM7_ESM.pdf]

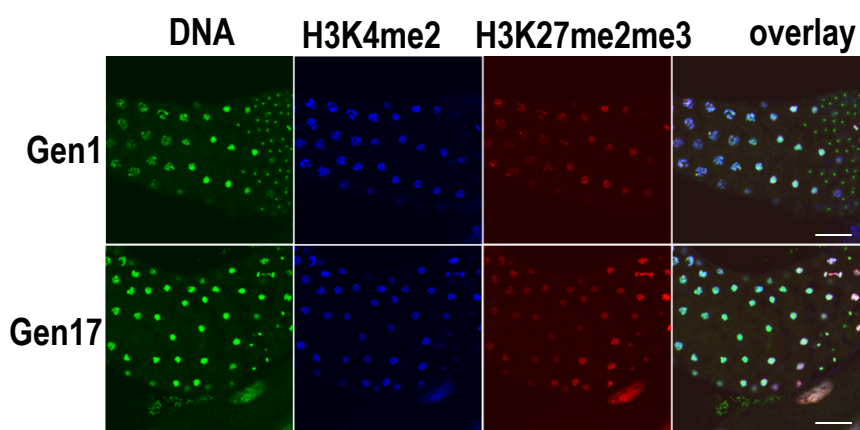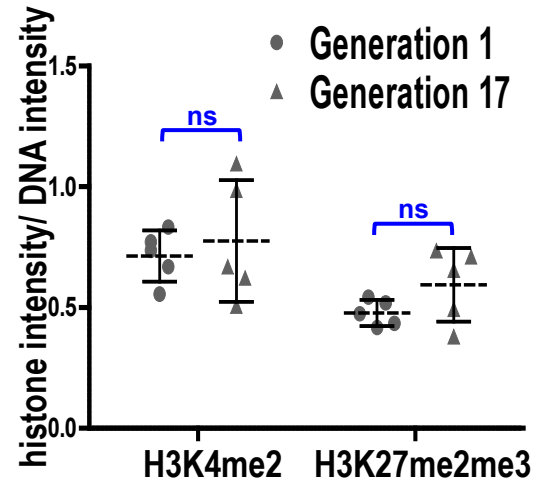

Supplement: Supplementary file 8 — Spermatocyte histone H3K4me2 and histone H3K27me2me3 appear similar between Generation 1 and Generation 17 males. Quantification of anti-H3K4me2 and anti-H3K27me2me3 in spermatocyte nuclei shows equivalent signal intensity of active (H3K4me2) and inactive (H3K27me2me3) histone marks in generation 1 (Gen 1) and Gen 17 male sup-46 mutants. Germlines were dissected, fixed, and stained with antibodies and PicoGreen (DNA). Scale bars represent 15 μm. Images are of a single confocal optical plane, with laser intensity and gain identical between samples. To ensure detection of the fluorescence signal was equivalent in spermatocytes that were on different focal planes, the histone signals were normalized to PicoGreen (DNA) intensity from the same spermatocyte. Hatched line and error bars are the mean ± SD (n = 5). ns, not significantly different (P > 05). (PDF 354 kb) [file 12915_2017_398_MOESM8_ESM.pdf]
