# Supplementary material for: C. elegans SUP-46, an HNRNPM family RNA-binding protein that prevents paternally-mediated epigenetic sterility
Source: BMC Biol. 2017 Jul 17;15:61. doi: 10.1186/s12915-017-0398-y (PMC5513350; doi:10.1186/s12915-017-0398-y)
Supplement: Supplementary file 9 — Additional methods. (DOCX 41 kb) [file 12915_2017_398_MOESM9_ESM.docx]

**Additional Methods:**

**Identification of *sup-46*:**

**Whole Genome Sequencing and PCR analysis of mutants:** To facilitate SNP mapping, we generated XA789 [*gna-2(qa705) unc-55(e1170) sup-46(qa710) unc-101(m1)I*]. We also used the following strains generated previously in our lab: DE12 [*sup-46(qa710)I*], XA774 [*gld-1(q485)/gna-2(qa705) unc-55(e1170)I*], XA792 [*sup-46(qa707)I*], XA796 [*gna-2(qa705) unc-55(e1170) sup-46(qa708)I],* XA797 [*sup-46(qa708)I*], or available from the Caenorhabditis Genetics Center (CGC; University of Minnesota, USA): AD189, AZ212, AZ244, DG3226, JH2099, N2 ("wild type"), RB656, RW10226. To prepare *sup-46(qa707)* DNA for whole genome sequencing, mixed stage worms were washed off NGM plates, purified by sucrose flotation (1), pelleted by centrifugation, and stored at -80^o^C until DNA extraction. To extract DNA, the pellet (~600 μl) was thawed and incubated for 30 minutes at 65^o^C in 10 ml Proteinase K SDS Buffer (100 mM Tris pH 8.5, 50 mM EDTA, 100 mM NaCl, 1% SDS, 1% β-mercaptoethanol, 400 μg/ml Proteinase K). Following digestion, the sample was extracted 2X with phenol chloroform and 1X with chloroform, precipitated in ethanol, washed 2X with 70% ethanol and redissolved in 1 ml Tris-EDTA buffer. A 500 μl aliquot was incubated with 2 μg RNase for 30 minutes at room temperature, and re-extracted as described above. DNA was recovered by spooling from an ethanol/buffer interface with a flame sealed glass Pasteur pipette, and the DNA was dissolved in 100 μl Tris-EDTA buffer.

**Confirming identification of SUP-46 by 1) RNAi phenocopy and 2) transgene rescue:** 1) *C25A1.4(RNAi)* suppresses *gna-2(qa705)* embryonic lethality: *Gna-2(qa705)* is 100% maternal effect embryonic lethal, so XA774 was used to test the effect of *sup-46(RNAi).* XA774 is a strain in which *gna-2(qa705)* is marked by *unc-55(e1170)* and balanced by *gld-1(q485)*, which is homozygous sterile with a tumorous germline*.* Larval stage L3/L4 worms were fed *empty vector(RNAi)* or *C25A1.4(RNAi)* (at 25^o^C) and their individual L3/L4 progeny were transferred to fresh RNAi plates (*empty vector(RNAi)*, n=42; *C25A1.4(RNAi)*, n= 45) . Subjecting worms to RNAi for two generations was sufficient to deplete SUP-46::FLAG::GFP from all tissues except neurons. Following two generations of RNAi, individual *gna-2(qa705) unc-55(e1170)* homozygous worms (Unc phenotype) were examined for the presence of progeny. 2) SUP-46::FLAG::GFP "rescues" (restores embryonic lethality) in *gna-2(qa705) unc-55(e1170) sup-46(qa708)*: DE103 (*unc-119(?) III; dnIs22 [unc-119(+) sup-46::flag::gfp*] was crossed with XA796, and Unc55 F_2_ were picked and examined for GFP and F_3_ viability. RNAi feeding bacteria for the following genes were acquired from the Ahringer/GeneService feeding library: *sup-46(RNAi)* (I-5G19), *csr-1(RNAi)* (IV-4E01), and control [*empty vector(RNAi)*] was made by transforming *E*. *coli* strain HT115(DE3) with *pPD129.36 (*a gift from Andy Fire, Stanford University, USA). All RNAi constructs were sequence-verified.

**RNASeq:**

Worms were purified by sucrose flotation. PolyA+ RNA was isolated using a QuickPrep micro mRNA Purification Kit (Amersham Biosciences). TruSeq RNA Sample Prep (Illumina) was used to re-purify mRNA for preparation of a barcoded cDNA library with average fragment size of 350-400 bp, using 1 µg of enriched mRNA and following manufacturer's instructions.The barcoded library was quality checked with Agilent Bioanalyzer and quantified by qPCR using KAPA SYBR FAST Universal Master Mix following Illumina’s Sequencing Library qPCR Quantification Guide (KapaBiosystem) running in Applied Biosystems 7900HT. The quality checked libraries were loaded on a flow cell for cluster generation using Illumina c-Bot and TruSeq PE Cluster v3.

**Microscopy and immunohistochemistry:**

Images in Figs. 3D (bottom row), 3G, and 3H were acquired using a NIKON Ti-E inverted confocal microscope (model Nikon C1si) fitted with a Plan ApoVC 60X oil immersion lens (aperture 1.4). Illumination was provided by 488nm Kr/Ar and 568 nm lasers, and detection by an Si spectral detector (400-750nm), and the acquisition software used was Nikon EZ-C1/Xp and Elements. For Fig. 3G, thirty 0.5μm optical sections were collected to generate a composite image that spanned the thickness of the embryo. The rest of the confocal imaging was performed using a Leica DMLSFA upright confocal microscope (model Leica SP2) fitted with a Leica HCX PL APO 40X oil immersion lens (aperture 1.25). Illumination was provided by 488 Kr/Ar, 543 and 633 diode lasers, and a user-controlled prism was used to select detection wavelength. Software utilized for image acquisition was Leica LCS/SP. Subsequent to image acquisition, Adobe Photoshop CS5 and Illustrator CS5 were used to stitch together overlapping images (Figs. 3A and Additional File 7B), to crop and compile images into Figure panels, and to place size bars.

The following primary antibodies (diluted in PBGS) were applied overnight at 4^o^C: rabbit polyclonal anti-GFP (1:200; Molecular Probes; Cat# A11122, Lot# 1356608; RRID: AB_221569), mouse monoclonal K76 anti-PGL-1 (1:40; Developmental Studies Hybridoma Bank; preparation date 11/12/09, 348 μg/ml; RRID: AB_531836), mouse monoclonal KT3 anti-PGL-3 (1:40; Developmental Studies Hybridoma Bank; preparation date 10/09/08, 24 μg/ml; RRID: AB_1556927), rabbit polyclonal anti-H3K4me2 (1:200; Millipore Sigma; Cat# 07-030, Lot# 2430486; RRID: AB_11213050), mouse monoclonal anti-H3K9me2 (1:100; Abcam; Cat# ab1220, Lot# GR183500-1; RRID: AB_449854), mouse monoclonal anti-H3K27me2me3 (1:300; Active Motif; Clone 7B11: Cat# 39535, Lot# 16714012), mouse monoclonal anti-SP56 (1:500; gift of S. Strome), and rabbit polyclonal anti-CGH-1(1:300; gift of K. Blackwell and P. Boag). Fluorescently-conjugated wheat germ agglutinin lectin (WGA), which we and others (2) have determined marks sperm membranous organelles (MO), was also used in some experiments to mark sperm. Secondary antibodies [Molecular Probes; diluted in PBGS; AlexaFluor GAM-546 (Cat# A11030, Lot# 1345046; RRID: AB_144695), AlexaFluor GAM-647 (Cat# A21235, Lot# 49625A; RRID: AB+141693), AlexaFluor GAR-488 (Cat# A11008, Lot# 459548; RRID: AB_143165), AlexaFluor GAR-546 (Cat# A11010, Lot# 84E2-1; RRID: AB_143156) were applied for 4 hours RT or overnight at 4^o^C, and SlowFade (Invitrogen) was added prior to addition of a coverslip and sealing of slides with nail varnish. In some experiments, DNA was marked with PicoGreen (1:200 in water, 10 μl added to slide prior to SlowFade) or the eggshell was marked with WGA-Alexa-TRITC, 647 (1:100, added with the secondary antibody).

**Immunoblotting:**

Eggs were harvested from gravid hermaphrodites by alkaline hypochlorite treatment (3), washed, pelleted by centrifugation and stored at -80^o^C. Frozen, packed egg pellets were transferred to liquid nitrogen and powdered using a Cellcrusher (Stratech Scientific Ltd., Suffolk ,UK). Samples of powder were examined microscopically to confirm mechanical disruption of eggs, and frozen powders were weighed and returned to -80^o^C. Samples were extracted in RIPA buffer (25mM Tris pH 8.0, 150 mM NaCl, 0.1% SDS, 0.5% Sodium deoxycholate and 1% Triton X) in a ratio of 20 µL buffer/1 mg sample powder, and debris was removed by centrifugation through an Ultrafree-MC VV Centrifugal Filter (Millipore Sigma) at 4^o^C. Protein was quantified using a Pierce BCA Protein Assay (ThermoFisher Scientific) and samples were diluted with RIPA and Laemmli Buffers to obtain a final protein concentration of 1µg/µL. Samples were heated to 70^o^C for 10 minutes and proteins were separated by SDS-PAGE (10% acrylamide; with a stacking gel) followed by transfer to Immobilon-P 0.45 µm transfer membrane (Millipore Sigma) using a Trans-Blot SD semi-dry transfer cell (BIO RAD). Membranes were blocked for 1 hour at room temperature in PBS with 0.5% Tween and 5% skim milk powder (PBSTM). Membranes were incubated overnight at 4^o^C with anti-GFP (1:1000 in PBSTM; Invitrogen; Cat# A-11122, Lot# 1356608; RRID: AB_221569), washed, and incubated for 1 hour at room temperature with goat anti-rabbit-HRP (1:1000 in PBSTM; Invitrogen; Cat#A16104, Lot# 38-1570031814; RRID: AB-2534776) followed by protein detection using SuperSignal West Femto Maximum Sensitivity Substrate (ThermoFisher Scientific). Blots were stripped with Restore Western Blot Stripping Buffer (ThermoFisher Scientific) and re-probed using anti-*C. elegans* γ-tubulin (1:1000 in PBGSM; gift of J. Powers and W. Saxton) followed by goat anti-rabbit-HRP and detection as described above.

**Proximity biotinylation coupled to mass spectrometry (BioID-MS):**

Flp-In T-Rex 293 cells were transfected using jetPRIME transfection reagent (Polyplus Cat# CA89129-924). Cells were seeded at 250,000 cells/well in a 6 well plate following manufacturer's protocol in 2 ml DMEM (Gibco Cat# 11995-065), supplemented with 5% FBS, 5% Cosmic calf serum and 100 U/ml Pen/Strep. The following day cells were transfected with 200 ng of pcDNA5-ProteinX-FLAG-BirA* and 2 µg of POG44 in 200 µl of jetPRIME buffer mixed with 3 µl of jet Prime reagent, as per the manufacturer's instructions. The next day, transfected cells were passaged to 10 cm plates and 24 hours later were selected for by the addition of hygromycin to the growth media to a final concentration of 200 µg/ml. Selection media was changed every 2-3 days until clear visible colonies were present, at which point cells were scaled up to the needed number of 150 mm plates. Cells were grown to 70% confluence before induction of protein expression using 1 µg/ml tetracycline, and media supplementation with 50 µM biotin for protein labeling. Cells were harvested 24 hours later as follows: media was decanted, cells were washed with 5 ml PBS per 150 mm plate and then harvested by scraping in 1 ml of ice cold PBS. Cells from 2 x 150 mm plates were pelleted by centrifugation and pellets were frozen on dry ice.

**Affinity purification and on bead digest:** Frozen cell pellets were lysed in 10 volumes of modified RIPA buffer (50 mM Tris-HCl pH 7.5, 150 mM NaCl, 1% Triton X-100, 1 mM EDTA, 1 mM EGTA, 0.1%SDS, Sigma-Aldrich protease inhibitors P8340 1:500, and 0.5% Sodium deoxycholate). After lysis, 1 µl of benzonase (250 U) was added to each sample and cell pellets were allowed to thaw/lyse completely with gentle mixing at 4^o^C (~20-30 minutes). Lysates were sonicated on ice at 65% amplitude using a Qsonica with a CL-18 probe and centrifuged at 4^o^C. After centrifugation, the supernatant was added to pre-washed streptavidin-sepharose beads (GE Cat# 17-5113-01; 30 µl bed volume per sample; washed with 1 ml RIPA buffer, minus protease inhibitors and sodium deoxycholate), and biotinylated proteins were affinity-purified at 4^o^C with gentle mixing (3 hours). After affinity purification, streptavidin sepharose beads were pelleted and the supernatant removed. Beads were washed by gentle pipetting twice in RIPA buffer (minus protease inhibitors and sodium deoxycholate), twice in TAP lysis buffer (50 mM HEPES-KOH pH 8.0, 100 mM KCl, 10% glycerol, 2mM EDTA, 0.1% NP-40), and 3 times in 50mM ammonium bicarbonate pH 8. After the last wash, residual 50 mM ammonium bicarbonate was removed and proteins were digested on bead. To digest, beads were resuspended in 30 µl of 50 mM ammonium bicarbonate pH 8 containing 1 µg of trypsin and incubated at 37^o^C overnight with gentle mixing. The next day an additional 0.5 µg of trypsin was added to each sample (in 10 µL 50 mM ammonium bicarbonate pH8) and samples incubated for an additional 2 hours at 37 ^o^C with mixing. Beads were pelleted by centrifugation, washed, and the supernatant transferred to a fresh 1.5 ml microfuge tube. Beads were rinsed 2 times with 30 µl of 0.2 µM filtered HPLC-grade H_2_O and pelleted by centrifugation and these rinses were combined with the original supernatant. The pooled supernatant was centrifuged and 80 µl were transferred to a new 1.5 ml microfuge tube, making sure to leave any beads behind. Samples were acidified with 2% formic acid and dried in a centrifugal evaporator.

**Mass Spectrometry:** Affinity-purified digested material from 2 x 150 mm plates was resuspended in 12 µl of 5% formic acid, centrifuged briefly and 6 µl was taken for MS analysis and 5 µl injected by autosampler onto a spray tip formed from a fused silica capillary column (0.75 µm ID, 350 µm OD) using a laser puller. The column was preloaded with 10 to12 cm of C18 reversed-phase material (ZorbaxSB, 3.5µm) by pressure bomb loading in MeOH and was pre-equilibrated with solvent A (100% H_2_O, 0.1% formic acid). The column was placed in-line with a LTQ-Orbitrap Velos (ThermoFisher Scientific) equipped with a nanoelectrospray ion source (Proxeon, ThermoFisher Scientific) connected in-line to a NanoLC-Ultra 2D plus HPLC system (Eksigent, Dublin, USA). The LTQ-Orbitrap Velos/Elite instrument under Xcalibur 2.0 was operated in the data dependent mode to automatically switch between MS and up to 10 subsequent MS/MS acquisition. The HPLC gradient program delivered an acetonitrile gradient over 125 minutes. For the first twenty minutes, the flow rate was 400 µl/min at 2% solvent B (100 ACN, 0.1% formic acid). The flow rate was then reduced to 200 µl/min and the fraction of solvent B increased in a linear fashion to 35% until 95.5 minutes. Solvent B was then increased to 80% over 5 minutes and maintained at that level until 107 minutes. The mobile phase was then reduced to 2% solvent B until the end of the run (125 minutes).

**MS/MS Data Analysis:** All Thermo RAW files were saved in our local interaction proteomics LIMS, ProHits (4). mzXML files were generated from ThermoFinnigan RAW files using the ProteoWizard (5) converter, implemented within ProHits (--filter “peakPicking true2” --filter “msLevel2”). The searched database contained the human and adenovirus complement of the RefSeq protein database (version 57) supplemented with ”common contaminants” from the Max Planck Institute (<http://141.61.102.106:8080/share.cgi?ssid=0f2gfuB>), the Global Proteome Machine (GPM; <http://www.thegpm.org/crap/index.html>), and common sequence tags. The sequence database consisted of forward and reversed sequences; in total, 72,226 sequences were searched. The search engines were Mascot and Comet, with trypsin specificity and two missed cleavage sites allowed. Methionine oxidation and asparagine/glutamine deamidation were set as variable modifications. The fragment mass tolerance was 0.6 Da and the mass window for the precursor was ±12 ppm. The resulting Comet and Mascot search results were individually processed by PeptideProphet (6), and peptides were assembled into proteins using parsimony rules first described in ProteinProphet (7) into a final iProphet (8) protein output using the Trans-Proteomic Pipeline (TPP; Linux version, v0.0 Development trunk rev 0, Build 201303061711). TPP options were as follows. For the Velos Orbitrap files, general options are -p0.05 -x20 -PPM - d"DECOY", iProphet options are pPRIME and PeptideProphet options are pPAEd. All proteins with a minimal iProphet protein probability of 0.05 were parsed to the relational module of ProHits. Note that for analysis with SAINT, only proteins with iProphet protein probability ≥ 0.95 are considered. This corresponds to an estimated protein level FDR of ~0.5%. A minimum of two unique peptide ions is also enforced. This data set consisting of 12 raw files and associated peak list and results files has been deposited in ProteomeXchange through partner MassIVE as a complete submission and assigned the MassIVE ID [MSV000080631] and [PXD006094], (ftp://massive.ucsd.edu/ MSV000080631).

**Significant Analysis of INTeractome (SAINT):** SAINT calculates, for each prey protein identified in a purification, the probability of true interaction by using spectral counting (semi-supervised clustering, using a number of negative control runs). SAINTexpress (9) analysis was performed using version exp3.3 with two biological replicates per bait. Bait protein samples were analyzed alongside 8 negative control runs [compressed to 4, as previously described (10)] consisting of purifications from untransfected cells or cells expressing BirA*, BirA*-FLAG, or BirA*-FLAG-GFP. Interactions with a calculated Bayesian FDR ≤ 1% were considered high confidence. Additional File 1: Table S2 shows the entire SAINT analysis with the exception of two preys that were manually removed: ENSBTAP00000016242 (a tubulin protein from Bovine) identified in HNRNPM screen and ENSEMBL:ENSBTAP00000032840 identified in MYEF2 screen. Visualization was with custom tools at ProHits-viz.lunenfeld.ca (“dotplot generator”, default options); once any protein passes the 1% FDR threshold in any bait-prey pair, all its quantitative values across all baits are recovered, irrespective of the individual FDR of the given bait-prey pair.

**References:**

1. Sulston J, Hodgkin J. Methods. In: Wood WB, editor. The Nematode *Caenorhabditis elegans*. Cold Spring Harbor: Cold Spring Harbor Laboratory; 1988. p. 587-606.

2. Ward S, Hogan E, Nelson GA. The initiation of spermiogenesis in the nematode Caenorhabditis elegans. Dev Biol. 1983;98(1):70-9.

3. Stiernagle T. Maintenance of C. elegans. WormBook. 2006:1-11.

4. Liu G, Zhang J, Larsen B, Stark C, Breitkreutz A, Lin ZY, et al. ProHits: integrated software for mass spectrometry-based interaction proteomics. Nat Biotechnol. 2010;28(10):1015-7.

5. Adusumilli R, Mallick P. Data Conversion with ProteoWizard msConvert. Methods Mol Biol. 2017;1550:339-68.

6. Keller A, Nesvizhskii AI, Kolker E, Aebersold R. Empirical statistical model to estimate the accuracy of peptide identifications made by MS/MS and database search. Anal Chem. 2002;74(20):5383-92.

7. Nesvizhskii AI, Keller A, Kolker E, Aebersold R. A statistical model for identifying proteins by tandem mass spectrometry. Anal Chem. 2003;75(17):4646-58.

8. Shteynberg D, Deutsch EW, Lam H, Eng JK, Sun Z, Tasman N, et al. iProphet: multi-level integrative analysis of shotgun proteomic data improves peptide and protein identification rates and error estimates. Mol Cell Proteomics. 2011;10(12):M111 007690.

9. Teo G, Liu G, Zhang J, Nesvizhskii AI, Gingras AC, Choi H. SAINTexpress: improvements and additional features in Significance Analysis of INTeractome software. J Proteomics. 2014;100:37-43.

10. Mellacheruvu D, Wright Z, Couzens AL, Lambert JP, St-Denis NA, Li T, et al. The CRAPome: a contaminant repository for affinity purification-mass spectrometry data. Nat Methods. 2013;10(8):730-6.
